# Locally Produced IL-10 Limits Cutaneous Vaccinia Virus Spread

**DOI:** 10.1371/journal.ppat.1005493

**Published:** 2016-03-18

**Authors:** Stephanie S. Cush, Glennys V. Reynoso, Olena Kamenyeva, Jack R. Bennink, Jonathan W. Yewdell, Heather D. Hickman

**Affiliations:** 1 Laboratory of Viral Diseases, National Institute of Allergy and Infectious Diseases, National Institutes of Health, Bethesda, Maryland, United States of America; 2 Biological Imaging Section, Research Technologies Branch, National Institute of Allergy and Infectious Diseases, National Institutes of Health, Bethesda, Maryland, United States of America; Thomas Jefferson University, UNITED STATES

## Abstract

Skin infection with the poxvirus vaccinia (VV) elicits a powerful, inflammatory cellular response that clears virus infection in a coordinated, spatially organized manner. Given the high concentration of pro-inflammatory effectors at areas of viral infection, it is unclear how tissue pathology is limited while virus-infected cells are being eliminated. To better understand the spatial dynamics of the anti-inflammatory response to a cutaneous viral infection, we first screened cytokine mRNA expression levels after epicutaneous (ec.) VV infection and found a large increase the anti-inflammatory cytokine IL-10. *Ex vivo* analyses revealed that T cells in the skin were the primary IL-10-producing cells. To understand the distribution of IL-10-producing T cells *in vivo*, we performed multiphoton intravital microscopy (MPM) of VV-infected mice, assessing the location and dynamic behavior of IL-10 producing cells. Although virus-specific T cells were distributed throughout areas of the inflamed skin lacking overt virus-infection, IL-10^+^ cells closely associated with large keratinocytic foci of virus replication where they exhibited similar motility patterns to bulk antigen-specific CD8^+^ T cells. Paradoxically, neutralizing secreted IL-10 *in vivo* with an anti-IL-10 antibody increased viral lesion size and viral replication. Additional analyses demonstrated that IL-10 antibody administration decreased recruitment of CCR2^+^ inflammatory monocytes, which were important for reducing viral burden in the infected skin. Based upon these findings, we conclude that spatially concentrated IL-10 production limits cutaneous viral replication and dissemination, likely through modulation of the innate immune repertoire at the site of viral growth.

## Author Summary

While ineffective antiviral immune responses can result in illness or even death, excessive host responses can also cause substantial injury. Anti-inflammatory proteins play an important regulatory role in limiting immune-mediated damage, but it is unknown where the cells making these modulators need to be for the greatest effect. The best-described immune-response-limiting protein is the cytokine interleukin-10 (IL-10), which is produced during infections with disparate pathogens including viruses, bacteria, and parasites. Despite the preponderance of IL-10 production during infection, we do not know the tissue distribution of this cytokine or whether it acts in localized areas. To address these questions, we studied the behavior of IL-10-producing cells after infecting mice with vaccinia virus (VV), the attenuated vaccine virus used to eradicate smallpox. Using microscopy to image infection in the skin of living mice, we found that IL-10 is produced by cells that surround areas of virus replication, rather than throughout the tissue as we anticipated. Even more surprisingly, we found that IL-10 promotes (rather than impedes) virus clearance, likely by shaping the tissue response of innate immune cells. These data show that supplying anti-inflammatory cytokines at precise areas of infected tissue can dramatically limit viral replication and damage.

## Introduction

Ideally, the antiviral immune response eliminates actively replicating virus and any viral reservoirs without undue host damage. For many viruses, however, the immune response extends beyond that necessary for viral clearance and creates disease symptoms. For example, infection with certain influenza virus (IAV) strains results in lung recruitment of high numbers of neutrophils (and other leukocytes) leading to a fast-progressing viral pneumonia and extensive lung damage [1, 2]. Neutrophil recruitment at late stages of infection can also lead to CNS pathology during coronavirus infection [3]. Virus-specific CD4^+^ T cells substantially contribute to respiratory syncytial virus (RSV)-induced bronchiolitis in children [4]. Further, early RSV vaccination strategies actually enhanced disease due to excessive cellular infiltration of the lungs and subsequent pulmonary injury [5]. Thus, the immune response, essential for eliminating pathogens, also produces disease if not appropriately modulated.

IL-10 is an important anti-inflammatory cytokine that quells innate and adaptive immune responses during both infection and autoimmunity [6–9]. Originally named “cytokine synthesis inhibitory factor,” IL-10 impedes the production of a number of pro-inflammatory cytokines and chemokines secreted by antiviral T cells for the control of infection, including IFN-γ, TNF-α, and MIP-1α [10, 11]. While IL-10 restrains host pathology caused by the immune response during acute infections with IAV, RSV, or coronavirus [12–14], it can also limit viral clearance leading to chronic infection [15–17]. Accordingly, several viruses have evolved homologs of IL-10 or its receptor (IL-10R) to manipulate the host immune environment and enable persistence [18–20].

Paradoxically, during some acute viral infections highly activated, pro-inflammatory CD8^+^ T cells can also produce IL-10 and even represent a major source of IL-10 in infected organs [21–24]. The role of such poly-secretory CD8^+^ T cells appears to be dictated by both viral tropism and the site of infection, but the principle function ascribed to these cells is suppression of inflammatory tissue damage rather than direct alteration of viral replication. For example: antibody (Ab) blockade of the IL-10 receptor (IL-10R) during IAV infection increases lung inflammation and mortality without reducing viral burden [21]. Similarly, RSV-activated IL-10^+^ CD8^+^ effector T cells prevent RSV-induced inflammatory lung pathology [23]. During coronavirus-infection, IL-10^+^ CD8^+^ T cells limit CNS demyelination and slightly increase viral burden as a necessary cost [12]. The extent to which localized IL-10 production by T cells occurs in other acute viral infections in organs more resilient to inflammatory damage is uncertain.

Multiple aspects of IL-10 production have been proposed to shape disease outcome, including IL-10 production kinetics and shifts in the anatomical location of IL-10 depots [9, 25]. The local distribution of IL-10 (or any other inflammatory cytokine) production *in situ* or the spatiotemporal contribution of IL-10-producing cells to viral clearance is unknown. Previously, we reported that during vaccinia virus (VV) infection of the skin, myeloid innate immune cells and CD8^+^ effector T cells exhibited strikingly different distributions [26]. Thus, the precise positioning and behavior of IL-10-producing cells could dramatically influence virus clearance in localized areas.

Here, we analyze the dynamics of IL-10-producing cells *in situ* after epicutaneous (ec.) VV infection. VV skin infection induces a large population of IL-10^+^ CD4^+^ and IL-10^+^ CD8^+^ T cells in the infected tissue. Intriguingly, IL-10^+^ T cells more closely localize to viral replicative foci than do the bulk population of antiviral CD8^+^ T cells in the tissue. Further, IL-10^+^ T cells move in paths adjacent to areas of virus infection, and neutralization of IL-10 enhances virus spread and shapes the innate immune population located adjacent to these foci. Together, our data demonstrate that spatially directed anti-inflammatory cytokine production inhibits viral dissemination within the infected tissue and illuminate a novel aspect of antiviral immunity.

## Results

### Epicutaneous VV infection elicits a robust IL-10^+^ T cell response

Multiple inflammatory immune effectors, including cytotoxic CD8^+^ T cells and innate Ly6G^+^ cells, synergize to control recombinant VV (rVV) infection of the skin [26]. Despite a highly inflammatory Th_1_-polarized cellular infiltrate, viral lesions after epicutaneous (ec.) infection of C57Bl/6 mice resolve in 14 days with limited, rapidly repaired tissue damage.

**Fig 1:**
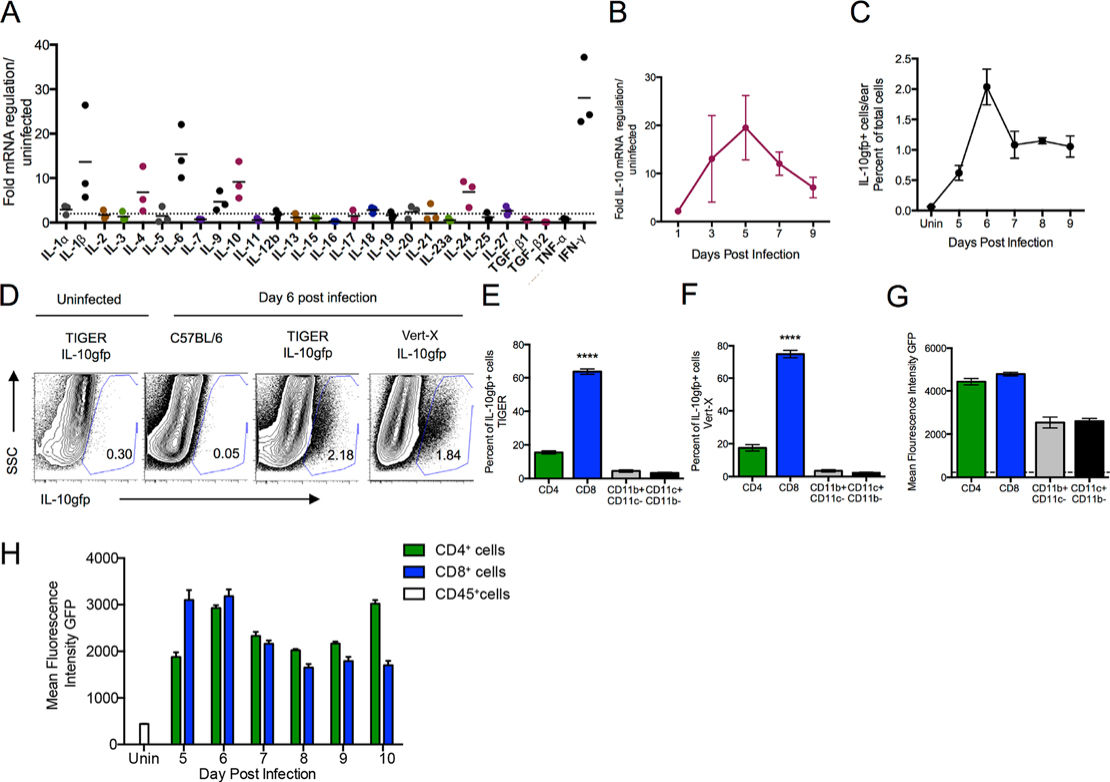
IL-10 is produced in VV-infected skin by T cells. **A)** Fold mRNA regulation of the indicated gene (x-axis) in VV-infected ears (6 d.p.i.) as quantified by real-time PCR. Dashed line demarcates a two-fold increase over uninfected ears. Dots represent separate ears. **B)** Time-course of IL-10 mRNA fold regulation. **C)** The mean frequency of GFP^+^ cells in the skin per day in IL-10gfp reporter mice (TIGER). N = 4 per time point, P< 0.0001 one-way ANOVA versus uninfected **D)** Representative FACs plots of TIGER or Vert-X mice 6 d.p.i. **E-F)** The relative contributions of different immune cell subsets to the total GFP^+^ population day 6 p.i. in TIGER (E) or Vert-X mice (F). N = 4 **G)** Geometric mean fluorescence intensity of GFP in indicated cell populations on day 6 p.i., N = 4. Dashed line indicates the MFI of GFP^-^ population. **H**) Timecourse of mean fluorescence intensity of GFP in either CD4^+^ cells (green bars) or CD8^+^ cells (blue bars). CD45^+^ cells were analyzed in uninfected mice due to the lack of T cells in uninfected N = 4. Error bars = SEM. **** = P < 0.0001.

To understand the potential mechanisms of immune self-restraint during ec. VV infection, we performed qPCR arrays, analyzing expression of cytokine mRNAs isolated from infected tissue at 6.5 days post-infection (d.p.i.) (Fig 1A). At this time, VV titers begin to wane in the skin concomitant with the entry of substantial numbers of virus-specific T cells [26]. qPCR revealed that VV-infection induces multiple pro-inflammatory cytokine mRNAs, including IL-1β, IL-6, and IFN-γ (black dots), but also surprisingly, IL-10 (magenta dots), which increased > 10-fold. Temporal mRNA analysis showed maximal IL-10 mRNA induction peaked around day 5 p.i. followed by a gradual decline (Fig 1B).

Using flow cytometry to monitor IL-10 production, we examined single cell suspensions derived from the ears of ec. VV-infected IL-10gfp reporter mice (“TIGER” (interleukin-ten IRES GFP-enhanced reporter mice)) [27] (Fig 1C–1H). Kinetic analysis identified GFP^+^ IL-10-producing cells in the skin by 5 d.p.i., peaking on day 6 at 2.2% of total cells, and then declining to a near constant level of ~1% of total cells until 10 d.p.i. (Fig 1C). Consistent with previous reports [27], we detected few (0.3%) GFP^+^ cells in uninfected reporter mice, or in infected wild-type mice (0.05% GFP^+^) (Fig 1D). We found similar frequencies of GFP^+^ cells (1.84%) using an alternative IL-10 reporter mouse (Fig 1D, far right panel) (Vert-X, also expressing IL-10-IRES-eGFP, but with a chimeric poly A tail sequence on transcripts [28] (see [29] for discussion of the properties of IL-10 reporter strains).

Surprisingly, the majority (> 65%) of IL-10gfp^+^ skin cells on day 6 post-recombinant vaccinia virus (rVV)-infection of TIGER mice were CD8^+^ T cells (Fig 1E), as defined by the cell-surface markers CD45, CD8α, and CD8β. CD4^+^ T cells comprised the second largest GFP^+^ population (~15%) on day 6, with minimal contributions from dendritic cells (DCs, CD11c^+^, CD11b^-^) or macrophages (CD11b^+^CD11c^-^). GFP^+^ populations were virtually identical in Vert-X reporter mice (shown 6 d.p.i., Fig 1F), confirming CD8^+^ T cells as the most prevalent IL-10-producing cells recovered from VV-infected skin on day 6 p.i. On a per-cell basis on day 6 p.i., CD8^+^ T cells expressed slightly higher levels of the GFP than CD4^+^ T cells, which were, in turn, higher than the very small number of GFP^+^ macrophages or DCs (Fig 1G). Over time, the mean fluorescence intensity of T cells was roughly equivalent, until CD4^+^ T cells acquired higher MFI at late times in infection (Fig 1H). We confirmed that both CD4^+^ and CD8^+^ T cells were producing IL-10 protein by intracellular cytokine staining (S1 Fig).

Together, these data show that ec. VV infection elicits a sizeable population of IL-10-expressing T cells in the skin over the course of acute infection, with IL-10^+^ CD8^+^ T cells being more numerous.

### IL-10gfp^+^ T cells accumulate in VV-infected tissue

**Fig 2:**
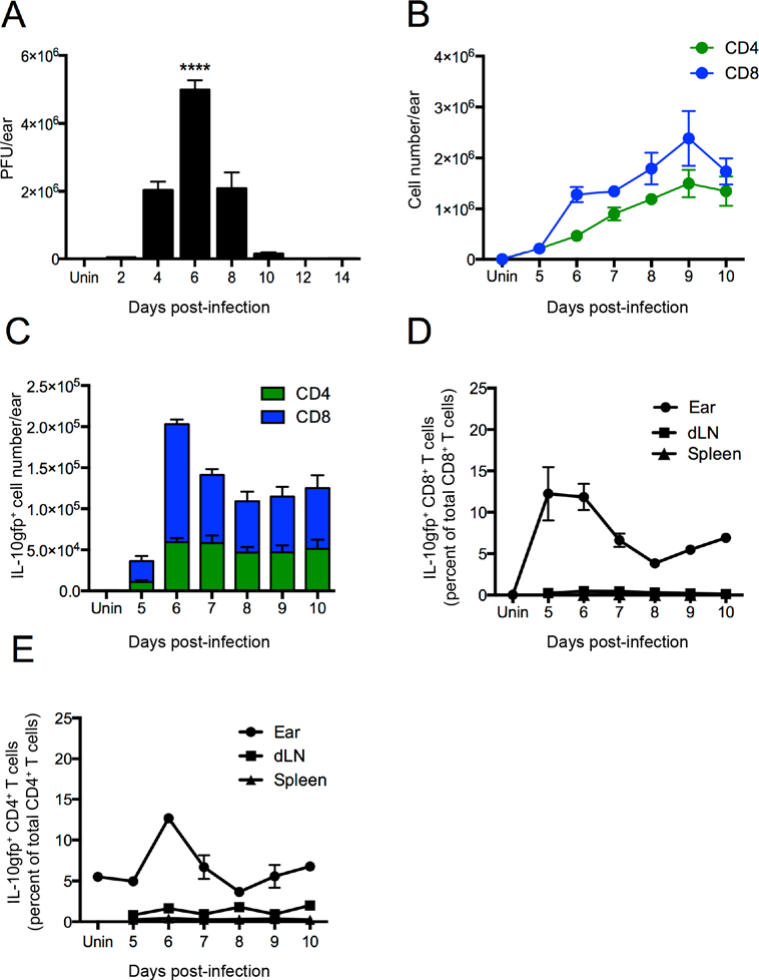
Kinetics of anti-VV IL-10^+^ CD8^+^ T cell responses. **A)** Viral titers after epicutanous infection of the ear as determined by plaque assay at indicated day p.i. N = 3 **B)** Time course of total CD4^+^ (green lines) and CD8^+^ T cells (blue lines) in the ear skin. N = 4. **C)** IL-10^+^CD4^+^ (green) and IL-10^+^CD8^+^ (blue) T cells. N = 4. **D)** Percentage of IL-10gfp^+^ CD8^+^ T cells of total CD8^+^ T cells in the ear skin (circle), draining lymph node (dLN, square), or spleen (triangle). **E)** Percentage of IL-10gfp^+^ CD4^+^ T cells of the total CD4 T cells as in (D). Error bars = SEM. **** = P <0.0001.

We next examined VV replication after ec. infection of the skin (Fig 2A). Viral titers peaked at 6 d.p.i. at ~ 5 x 10^6^ plaque forming units (PFU)/ear and dropped to close to the limit of detection by 10 d.p.i. Using flow cytometry, we enumerated total CD4^+^ and CD8^+^ T cells in ear single cell suspensions over the course of infection (Fig 2B). Few T cells were present in uninfected skin. Recombinant VV infection recruited T cells to the skin beginning at 5 d.p.i., with ~2 x 10^5^ cells/ear, peaking 4 days later at > 10^6^ each of CD4^+^ and CD8^+^ T cells per ear. Throughout the course of infection, CD8^+^ T cells were more prevalent than CD4^+^ T cells. Unlike total T cell numbers, IL-10gfp^+^ CD8^+^ T cells peaked soon after initial migration into infected tissue at 6 days p.i. (1.5 x 10^5^ cells/ear (Fig 2C)). Notably, IL-10gfp^+^ CD8^+^ T cell numbers declined within a day to a lower constant level, while IL-10gfp^+^ CD4^+^ T cells reached peak values at day 6 and remained relatively constant (average 5.8 x10^4^/ear daily).

To determine whether IL-10^+^ T cells were restricted to the infected tissue, we analyzed the spleen and draining lymph nodes (dLNs) from VV-infected animals (Fig 2D and 2E). We detected almost no IL-10gfp^+^ CD8^+^ T cells outside of the ear, despite the presence of highly activated CD8^+^ T cells in the spleen or node, demonstrating that IL-10gfp production is not inextricably linked to VV-induced T cell activation. A small and steady population of IL-10gfp^+^ CD4^+^ T cells (1 x 10^5^ cells) was present in DLNs, likely representing regulatory T cells [30] (Fig 2E).

Thus, at peak, between 10 and 15% of T cells express IL-10 in the skin during VV infection.

### VV-elicited IL-10gfp^+^ T cells are highly activated effectors

**Fig 3:**
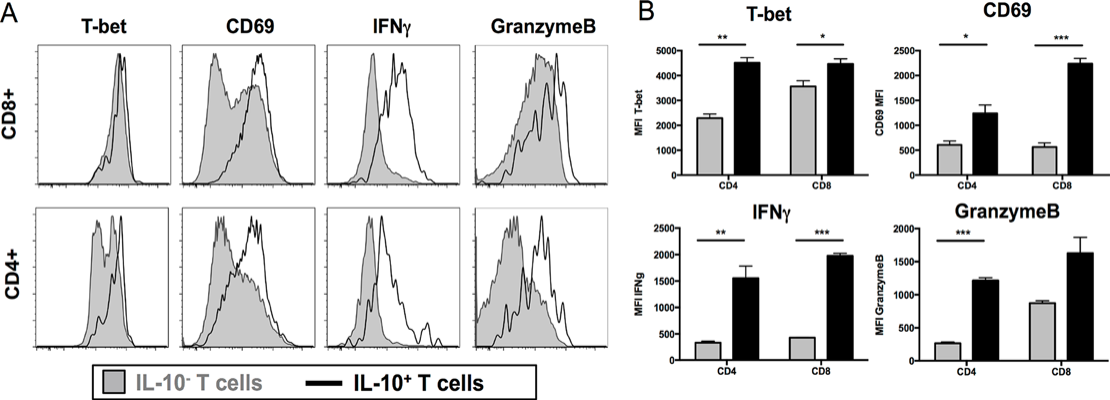
IL-10gfp^+^ T cells are highly activated. **A)** Flow histograms for the indicated markers (top legends) on day 6 p.i. gated on CD45^+^ CD8^+^ T cells (top panels) or CD45^+^ CD4^+^ T cells (bottom panels), followed by gating on the IL-10^+^ (black lines) or IL-10^-^ (gray fill) cells. **B)** Geometric MFI of markers in (A) for IL-10^-^ (gray bars) or IL-10^+^ (black bars) cells. Error bars = SEM. N = 4. * = P < 0.05, ** = P <0.01, *** = P <0.001.

Remarkably, IL-10^+^ CD8^+^ T cells responding to IAV, RSV, or coronavirus infection co-produce a number of pro-inflammatory effector cytokines [12, 21]. We examined skin T cells present at 6 days p.i. for expression of gene products associated with activated effector cells. All skin-localized CD8^+^ T cells expressed T-bet (a transcription factor required for effector T cell differentiation [31, 32]), regardless of IL-10 expression (determined by intracellular staining, gray shaded histogram = IL-10^-^ cells, black line = IL-10^+^ cells) (Fig 3A, far left, top panel). In contrast, while IL-10^+^ CD8^+^ T cells nearly uniformly expressed high levels of T-bet, total CD4 T cells contained both T-bet^high^ and T-bet^low^ cells (Fig 3A, far left, bottom panel). While there were clear populations of CD69^high^ and CD69^low^ IL-10^-^ T cells, all IL-10^+^ T cells expressed high levels of the activation marker CD69 (Fig 3A). In both CD4^+^ and CD8^+^ T cells, effector molecules IFN-γ and Granzyme B were markedly higher in IL-10^+^ cells (Fig 3B). We did not detect regulatory CD8^+^ T cells in the ear (S2 Fig). Thus, both IL-10^+^ CD4^+^ and CD8^+^ T cells in VV-infected skin are activated effectors.

### IL-10gfp^+^ T cells accumulate around VV keratinocytic foci

**Fig 4:**
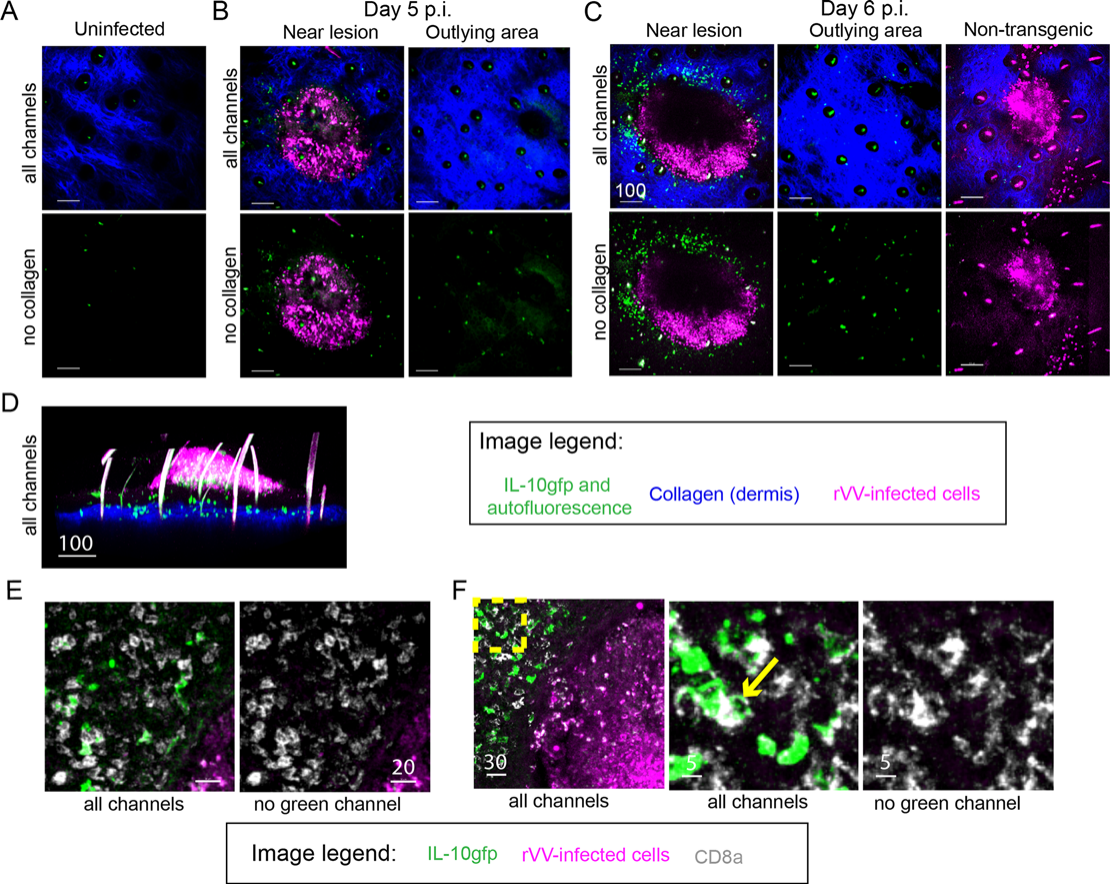
IL-10gfp^+^ CD8^+^ T cells surround viral lesions. Maximum intensity projections (MIP) of MPM image of ears from IL-10gfp reporter mice infected for the indicated times (below). Top panels show all channels, bottom panel shows no collagen (blue) channel for easier viewing of GFP signal. Collagen = blue, rVV-infected cells = blue pseudocolored magenta, IL-10gfp^+^ cells = green. **A)** Uninfected skin. **B)** Day 5 p.i. with rVV-NP-S-BFP. Outlying area is devoid of virus-infected cells to illustrate distribution of IL-10gfp^+^ cells. **C)** Day 6 p.i. with rVV NP-S-BFP as in (B). Non-transgenic is shown for background levels of GFP in infected mice (right panel). **D)** MPM MIP sideview (x-z axis) of a VV lesion at day 6 p.i. Note hairs (spikes seen due to MP excitation) in magenta channel. **E)** Confocal images from two different fields of frozen sections 6 d.p.i. with VV-NP-S-BFP (pseudocolored magenta), CD8α staining (gray), and IL-10gfp (green). **F)** Yellow box shows zoomed area; arrow points to punctate CD8α staining. Scale bars = microns.

We next used intravital MPM to directly visualize the location and movement of IL-10gfp^+^ cells in VV-infected reporter (TIGER) mice. Consistent with *ex vivo* experiments, we detected few IL-10gfp^+^ cells (green) in uninfected skin (note that we can visualize the dermis through the second harmonic excitation of collagen (blue) [33]) (Fig 4A). Five d.p.i. with an rVV expressing nuclear-targeted BFP to enhance discrimination of infected cells (VV-NP-S-BFP [34]), we consistently observed a population of IL-10gfp^+^ cells (green) surrounding keratinocytic foci of viral replication (pseudocolored magenta) (note that hairs are also excited by the MP laser and appear green within black follicles in the skin) (Fig 4B, left panels). Cellular GFP expression in tissue from areas devoid of VV-infected cells was similar to that in uninfected animals, despite virus-induced inflammation (Fig 4B, right panels).

Consistent with flow cytometric results, by 6 d.p.i., numerous IL-10gfp^+^ cells were recruited around VV replicative foci (Fig 4C, left panels). Outlying areas contained only slightly more IL-10gfp^+^ cells on day 6 p.i. than day 5 p.i. (Fig 4B and 4C). Computer generated maximum intensity projection (MIP) reconstructions clearly locate the bulk of IL-10gfp^+^ cells in the dermis below VV lesions, with a few IL-10gfp^+^ cells managing to enter lesions and interact with BFP^+^ infected cells (Fig 4D and S1 Movie).

To confirm that peri-lesional GFP^+^ cells represent IL-10gfp^+^ T cells detected *ex vivo* in dissociated skin suspensions, we stained frozen tissue sections taken 6 d.p.i. with anti-CD8 Ab (CD8α, white) (Fig 4E). Near lesions, we detected high numbers of CD8^+^ T cells, consistent with our previous description of CD8^+^ T cell accumulation near viral lesions [34]. Intriguingly, nearly all GFP^+^ cells surrounding viral foci expressed CD8, often in a punctate pattern reminiscent of Ag-engagement-induced TCR clustering [35] (for example, see Fig 4F, yellow arrow).

Taken together, these data show that IL-10gfp^+^ T cells strongly localize at the perimeter of VV-infected keratinocytes.

### IL-10gfp^+^ T cells closely associate with viral lesions in the skin

**Fig 5:**
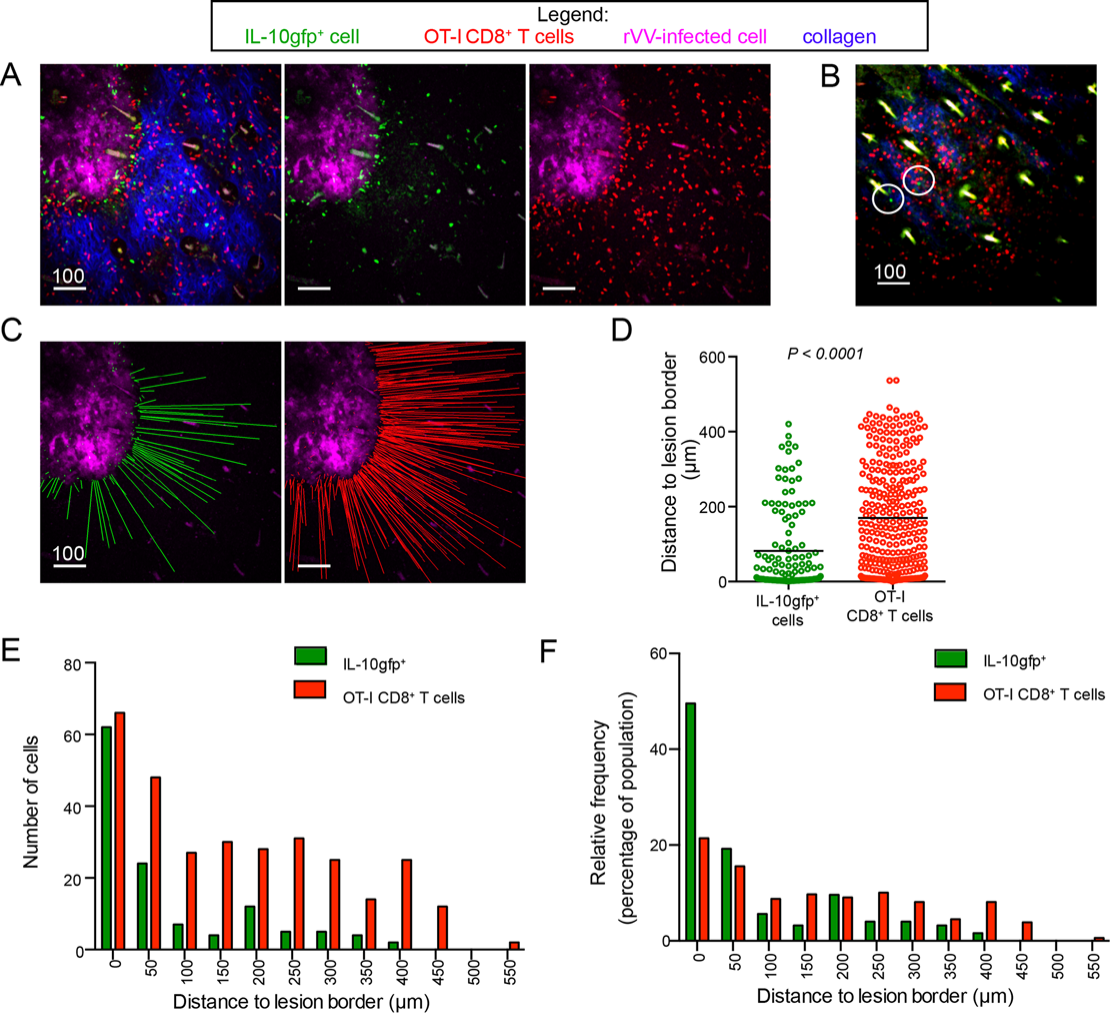
IL-10gfp^+^ CD8^+^ T cells closely localize to areas of virus infection. Maximum intensity projections (MIP) of MPM image of ears from IL-10gfp reporter mice taken on day 6 pi. Collagen = blue, rVV-infected cells = blue pseudocolored magenta, IL-10gfp^+^ cells = green, OT-I CD8^+^ T cells = red. **A**) MIP MPM images showing distribution of —10gfp+ T cells (green) versus non-IL-10gfp-transgenic OT-I CD8^+^ T cells (red). Left panel = merge, middle panel = only green and “virus” channels, right panel = only red and “virus” channels. **B**) As in A) but showing an outlying area without rVV-infected cells. Circles indicate IL-10gfp^+^ cells. **C**) Lines showing the distance of each cell to the border of the area of rVV-infection. Green lines show distance of IL-10gfp^+^ cells; red line OT-I CD8^+^ T cells. **D**) Calculated distance of each cell type to rVV lesion border. Line = mean. Statistics = student’s two-tailed t-test. **E**) Number of cells at each distance to the lesion border for each class of T cells. **F**) Percentage of each T cell class at indicated distance to lesion border. Scale bars = microns.

To further understand the localized delivery of IL-10 near viral replicative foci *in vivo*, we MPM imaged IL-10gfp^+^ cells in reporter mice over time (Fig 5). Using MPM imaging, we previously described the location and mobility of endogenously activated virus-specific T cells in the skin after ec. VV-infected mice [26]. Although all CD8^+^ T cells that immigrated into infected skin were Ag-specific effectors, many cells did not accrue at the border of lesions but instead occupied areas of the tissue that were not overtly infected as determined by virus-driven fluorescence expression [26]. Since we did not observe this pattern in IL-10gfp^+^ reporter mice (in which GFP-producing cells appeared to predominantly localize to lesions), we next used MPM imaging to compare the distribution of IL-10gfp^+^ cells to adoptively transferred virus-specific T cells endogenously activated in IL-10gfp-reporter mice (Fig 5). We first transferred non-IL-10gfp^+^ dsRed^+^ OT-I CD8^+^ T cells (antigen specificity = K^b^-SIINFEKL) into naïve IL-10gfp reporter mice, then infected ec. On day 6 p.i. (rVV expression of BFP is pseudocolored magenta), we detected both IL-10gfp^+^ cells (green) and IL-10gfp^-^/^-^ adoptively transferred OT-I CD8^+^ T cells (red) at the border of viral lesions (Fig 5A and S2 Movie). We also consistently observed areas of the skin distal from viral keratinocytic lesions with heavy infiltration of dsRed^+^ OT-I CD8^+^ T cells, but no or few IL-10gfp^+^ cells (Fig 5B and S3 Movie).

Calculating the distance of either cell type to the closest micrograph voxels containing viral fluorescence revealed that although both cell populations accrued near VV-infected foci, the population of IL-10gfp^+^ cells was significantly closer (81.6 ± 9.8 μm *vs*. 169.6 ± 8.1 μm from lesion border) (Fig 5C and 5D). Further, almost half of IL-10gfp^+^ cells were located within 50 μms of a lesion, whereas only 20% of dsRed^+^ OT-I CD8^+^ T cells (IL-10gfp^-^/^-^) were present in the same area (Fig 5E and 5F). Thus, unlike the majority of virus-specific CD8^+^ T cells recruited to the skin post-VV infection, on day 6 post-infection IL-10gfp^+^ cells are largely restricted to areas adjacent to viral lesions.

### IL-10gfp^+^ cells are mobile within a localized area

**Fig 6:**
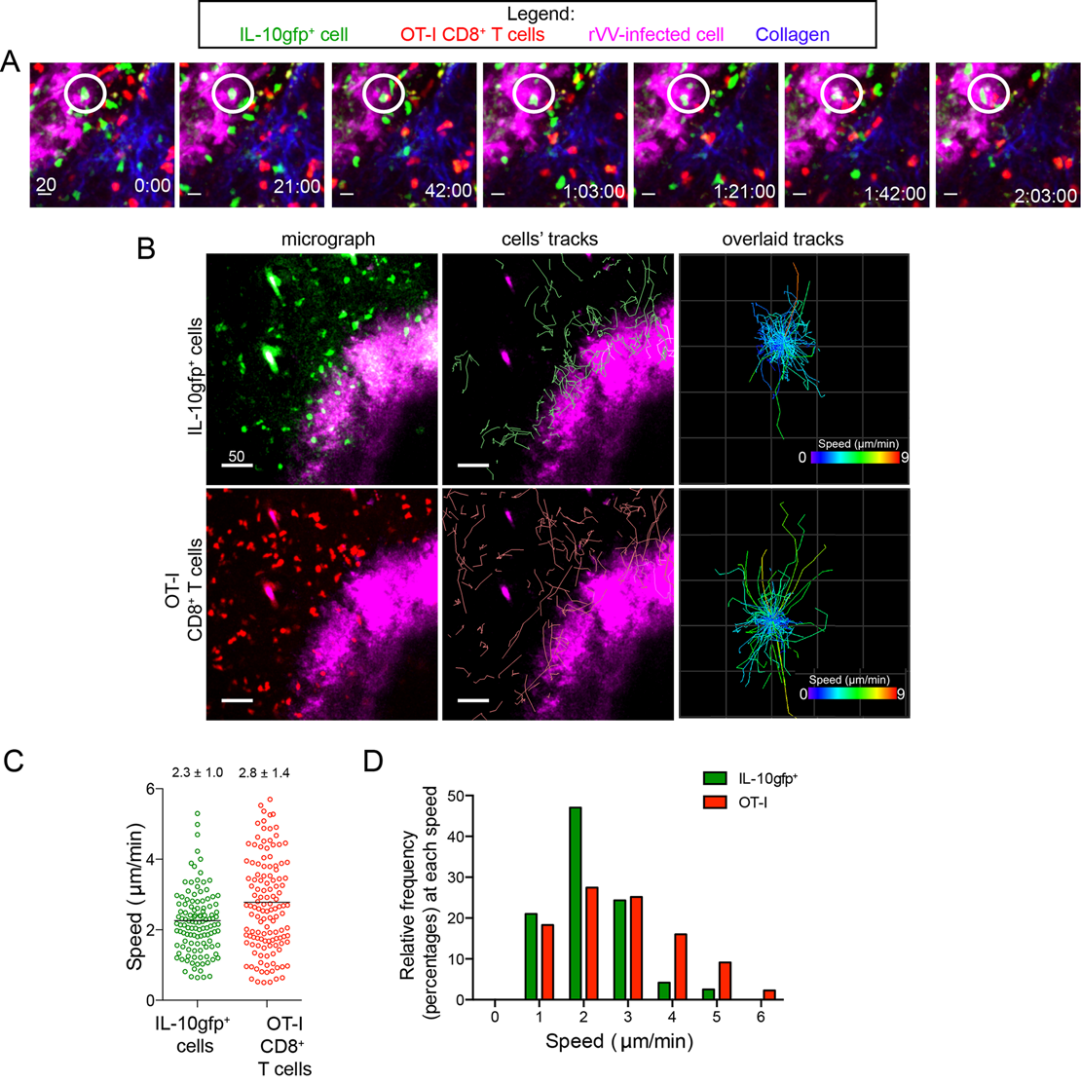
IL-10gfp^+^ CD8^+^ T cells are slowly motile around VV-lesions. **A-B)** Maximum intensity projections of MPM images taken 6 d.p.i. IL-10gfp^+^ cells = green, OT-I CD8^+^ T cells = red, rVV-infected cells = blue pseudocolored magenta, collagen = blue. **A**) Time-lapse images following an individual IL-10gfp^+^ cell (highlighted by white circle) that remains within a skin lesion during the entire 2 hr imaging period. Time (top of panels) is in minutes. **B)** Image (left) or tracks (middle) showing movement of IL-10gfp^+^ cells (top panel) or CD8^+^ OT-I cells (bottom panels) over an imaging period. Right panels show all tracks of either group of cells overlaid to a single starting point. **C)** Mean speeds of cells during one representative imaging session. Line = mean speeds; numbers indicate mean ± the SEM. **D)** Percentage of the total population of each cell type moving at the indicated speed. Green bars = IL-10gfp^+^ T cell, red bars = OT-I CD8^+^ T cells.

While static images revealed that IL-10-producing cells were located near areas of virus-infected cells, they could be 1) engaged and motionless, 2) motile at the perimeter of infection, or 3) only transiently associated with the borders of lesions. To examine these possibilities, we analyzed the movement of peri-lesional IL-10gfp^+^ cells in relation to dsRed^+^ OT-I CD8^+^ T cells within the same imaging field (Fig 6). Intriguingly, we noted both sessile and motile IL-10gfp^+^ cells at lesional borders during two-hour imaging sessions (Fig 6A and S4–S6 Movies). Calculating the tracks of each cell’s movements during this period revealed that mobile IL-10gfp^+^ cells followed paths near the outer border of viral lesions, remaining proximal to areas of viral infection (Fig 6B). We did not visualize IL-10gfp^+^ cells migrating away from infected areas during the course of imaging. An overlay of the tracks of all of the IL-10gfp^+^ cells clearly showed that IL-10gfp^+^ cells exhibited less overall displacement than did dsRed^+^ OT-I CD8^+^ T cells (Fig 6B, right panels).

We next tracked cellular speeds over the course of MPM imaging. Overall, peri-lesional IL-10gfp^+^ cells moved at slightly lower mean speeds than dsRed^+^ OT-I CD8^+^ T cells (mean speed of ~2.3 vs. 2.8 μm/min) (Fig 6C), though individual cells ranged in speed from nearly sessile to > 4 μm/min. A majority (~70%) of IL-10gfp^+^ cells were motile (defined by a speed > 2.5 μm/min) (Fig 5D). The bi-phasic behavior of cellular movement was confirmed by performing 7 independent experiments (S3 Fig).

Together, these data show that most IL-10gfp^+^ cells are mobile in confined areas around keratinocytic foci of infection.

### Localized IL-10 production affects tissue monocyte accumulation at infection sites

**Fig 7:**
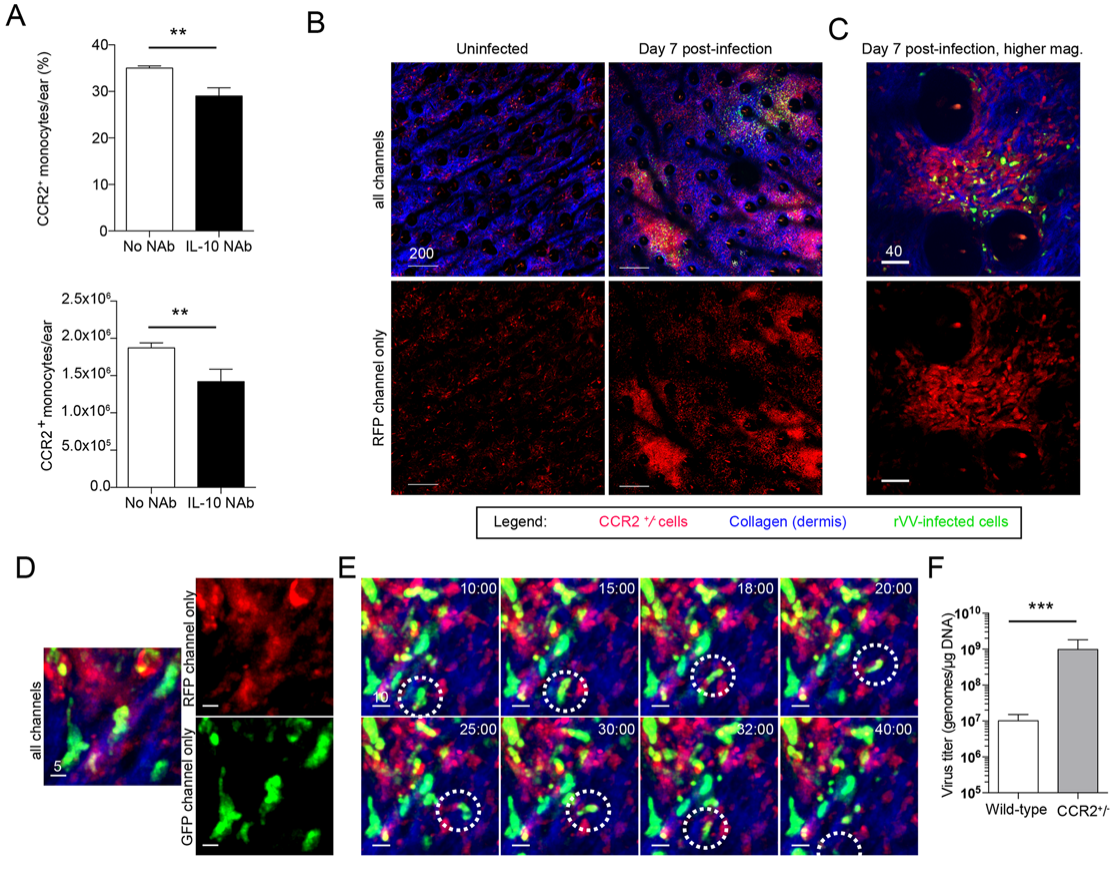
IL-10 neutralization alters tissue monocyte numbers. **A**) Percent (top, *p = 0*.*009*) and number (bottom, *p = 0*.*03*) of CCR2^+^ monocytes per ear on day 7 p.i. in either control mice or in mice given IL-10 neutralizing Ab (IL-10 NAb, black bars) on days 5 and 6 p.i. **B-C**) MPM images of CCR2rfp^+^/^-^ mice (with red monocytes) either uninfected (left panels) or 7 d.p.i. (right panels) with VV-NP-S-eGFP (green). For clarity, red channel only is shown in bottom panels. **D**) GFP^+^ (green) infected CCR2rfp^+^ monocytes. For clarity, single channels are shown. **E**) Timecourse MPM images showing movement of an infected (green) monocyte (red). Dashed circles follow one such infected cell. Time = min. **F**) Viral titers in CCR2rfp^+^/^-^ or control mice 7 d.p.i. (in genomes/μg of ear DNA). N = 3, *p = 0*.*0004*. Scale bars = microns.

Monocytes are recruited to and limit viral titers in VV-infected skin [26]. To analyze whether localized IL-10 production could affect the recruitment of these innate immune effectors, we administered anti-IL-10 neutralizing Ab (NAb) to wild-type mice on 5 and 6 d.p.i. This allowed IL-10 neutralization only after T cells had entered the tissue and eliminated possible confounding early effects on innate immunity (as could occur in IL-10 ^-^/^-^ animals). On day 7 p.i., we detected a significantly decreased number and percentage of CCR2^+^ tissue monocytes in the ears of IL-10-neutralized animals (Fig 7A).

We next MPM imaged CCR2^+^ tissue monocytes in heterozygous knock-in animals expressing RFP (in place of CCR2 protein) [36] (Fig 7B). We chose heterozygote mice to avoid complete deletion of CCR2^+^ cells (S4 Fig). In uninfected animals, we detected low levels of CCR2rfp^+^ cells in the ear (red, left panels). In contrast, VV infection resulted in dramatic accumulation of CCR2rfp^+^ cells in areas with viral-driven GFP expression (right panels and Fig 7C). We also detected a number of CCR2rfp^+^ cells that were directly infected with VV, as determined by GFP expression (Fig 7D and 7E). Many of these infected monocytes were motile (Fig 7E and S7 Movie). Further, CCR2rfp^+^/^-^ mice had dramatically increased VV titers in the ear compared to wild-type controls (Fig 7F).

Together, these data suggest that locally produced IL-10 enhances the recruitment of CCR2^+^ monocytes, which localize to areas of viral infection and reduce viral titers in the skin.

### Localized IL-10 production inhibits virus spread

**Fig 8:**
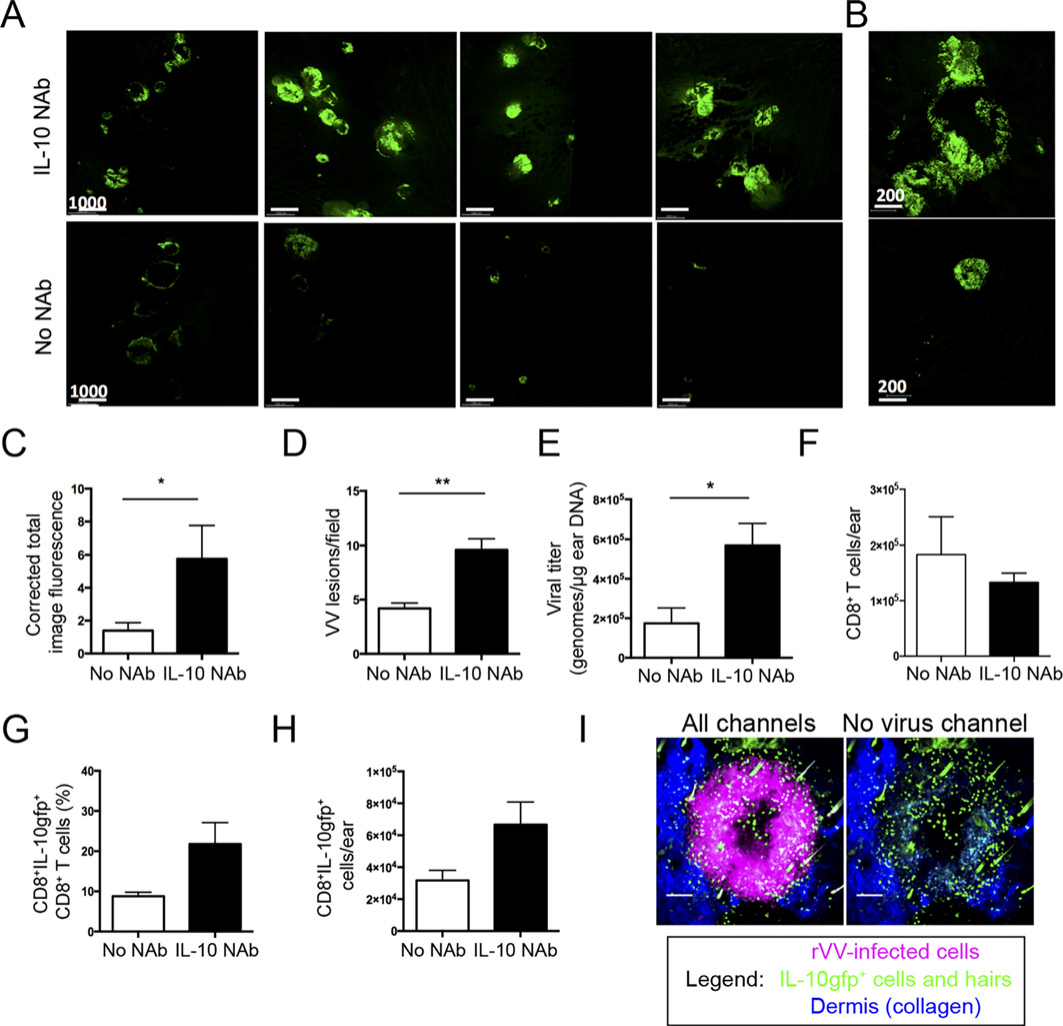
IL-10 limits viral replication and dissemination. **A)** Confocal image montages showing entire ears on day 7 p.i. with rVV-NP-S-eGFP (green) from IL-10 NAb (top) or non-NAb control (bottom). Each image is of a different ear. **B)** Higher magnification of confocal tile image as in (A). **C)** Total image fluorescence in confocal tile scans. N = 7, p = 0.03. **D)** Number of fluorescent VV lesions/7mm^2^ area, N = 7, p< 0.01. **E)** Number of VV genomes/μg of input DNA using qPCR, N = 6, p = 0.015. **F)** Total number of CD8^+^ T cells in the ear in IL-10-neutralized (black bars) and control (white bars) animals. N = 6. **G**) Percentage of IL-10gfp^+^ CD8^+^ cells in total CD8^+^ T cell population. N = 6 ± SEM. **H)** Absolute number of IL-10gfp^+^ CD8^+^ T cells in IL-10-neutralized (black bars) and control (white bars) animals, N = 6 ± SEM. **I)** MIP MPM image D7 p.i. of IL-10-neutralized animal. Left panel shows same image without magenta virus signal for easier discrimination of IL-10gfp^+^ cells (green). Error bars = SEM. Scale bars = microns.

To determine whether localized IL-10 production could shape tissue monocyte recruitment and affect VV clearance in the skin, we again administered anti-IL-10 NAb to wild-type mice on 5 and 6 d.p.i. and assessed the effect on virus replication and dissemination in the skin. Confocal microscopic montages of complete ears indicated an increase in VV-encoded fluorescence intensity and larger areas of fluorescence in anti-IL-10 NAb-treated mice (Fig 8A). Closer examination revealed that many of the VV lesions in IL-10-neutralized mice exhibited “satellites” indicative of secondary virus spread [37] (shown in higher magnification in Fig 8B). Quantitating total montage fluorescence revealed a 3-fold increase in VV-expressed GFP signal in IL-10 neutralized animals (Fig 8C). Likewise, the average number of VV lesions per (7x7 mm^2^ mosaic) image increased 2.3-fold (4.2 to 9.6) in IL-10-neutralized ears (Fig 8D). Importantly, real time PCR analysis of VV genomes present in the skin showed a concomitant 3-fold increase in virus (1.7 x 10^5^ to 5.7 x 10^5^ VV genomes/μg DNA) after IL-10 neutralization (Fig 8E).

Despite increased viral load, IL-10 neutralization did not dramatically alter the overall number of CD8^+^ T cells in the skin (Fig 8F). Intriguingly, neutralization trended toward an increased frequency and number of IL-10gfp^+^ CD8^+^ T cells in the skin (Fig 8G and 8H). In IL-10-neutralized animals, the enhanced number of IL-10gfp^+^ cells again localized to areas of virus replication (Fig 8I).

Based on these findings, we conclude that locally produced IL-10 limits skin VV-replication and dissemination. This extends the overly simplistic characterization of IL-10 as an “anti-inflammatory” cytokine, to include a direct antiviral role in immunity to pathogens.

## Discussion

Optimally resolving acute viral infections is a highly choreographed dance between effector cells that eliminate virus infection and anti-inflammatory cells that limit effector behavior to prevent excessive tissue damage. Much remains to be learned about how these two processes occur *in vivo* during viral infections. Currently, only a handful of studies have visualized the kinetics and behavior of virus-specific effector CD4^+^ or CD8^+^ T cells *in vivo* [34, 38–40]. The spatiotemporal behavior of cells secreting anti-inflammatory cytokines has not previously been analyzed during viral infection.

To begin to address these issues crucial to understanding antiviral immunity, we examined the production of the well-known anti-inflammatory cytokine IL-10 during rVV infection—including real time visualization of IL-10-producing cells in infected skin. We uncovered an unlikely primary cellular source of this cytokine: highly activated effector T cells (Figs 1–3) localizing to the immediate vicinity of intense virus replication (Fig 4). Remarkably, although IL-10 production should limit the ability of T cells to kill virus-infected cells, Ab-mediated neutralization of IL-10 actually increased viral titers in the skin.

Despite intense study, knowledge of all of the cells and immune factors necessary for VV clearance from the skin is still incomplete. After epicutaneous infection, a complex, spatially organized immune response resolves virus infection [34]. While T cells clear infected inflammatory monocytes from the skin, Ly6G^+^ monocytes also eliminate infectious virus. It is likely that many other immune cells types contribute synergistically to viral clearance. Here, we show that CCR2^+^ monocytes associate closely with virus-infected cells in the skin (even becoming infected (S7 Movie)), and also clearly aid in viral control (Fig 7). Interestingly, nitric oxide has been shown to prevent productive replication of VV in some macrophages [41]. Thus, CCR2^+^ monocytes could limit virus spread by “sopping up” infectious virus and preventing infection of cell types capable of propagating new virions.

Because IL-10 shapes macrophage populations in the tissues [42–44], we neutralized IL-10 and examined the effect on CCR2^+^ monocytes. Administration of IL-10 neutralizing Ab resulted in an approximately 25% reduction in the number of CCR2^+^ monocytes in the skin (Fig 7A). Altogether, our data strongly suggest that IL-10 reduces recruitment or maintenance of CCR2^+^ monocytes around sites of VV replication in the skin, which in turn results in failure to adequately control VV replication and dissemination.

IL-10 could participate in restriction of VV replication in the skin via other mechanisms besides CCR2^+^ monocyte recruitment. M2-polarized (also known as “alternatively activated” or “wound healing”) macrophages promote tissue restoration post-trauma, infection, or allergic inflammation [45–48]. Because VV has a proclivity for replicating/disseminating in inflamed skin (*e*.*g*. in individuals with atopic dermatitis [49, 50]), IL-10 could also influence VV replication by reducing VV-induced inflammation. Darling *et al*. recently used tape stripping and ovalbumin-sensitization to create atopic dermatitis-like skin lesions and then infected mice with VV [51]. IL-10 knockout mice had enhanced viral replication and skin lesions in an IL-17-dependent manner. Thus, IL-10 may also limit VV-replication by suppressing Th_17_ responses.

Additionally, IL-10 may directly inhibit VV replication by inducing innate anti-viral pathways in infected cells. An early study characterizing an IL-10-overexpressing rVV revealed no effect on viral replication, however IL-10 may already be saturating in wild-type mice [52]. Intraperitoneal infection of IL-10 ^-^/^-^ mice or Ab-mediated IL-10R blockade decreased ovarian VV titers slightly at 7 d.p.i. [53, 54]. Byrd and colleagues recently reexamined VV replication in human macrophages (previously described as non-permissive for productive VV replication [55, 56]) differentiating them into M1 and M2 polarized phenotypes [57]. VV replicated in both cell types, and importantly, the addition of exogenous IL-10 dramatically decreased VV replication in M2 macrophages in a STAT3-dependent manner. These findings suggest that IL-10 may regulate VV replication by inducing an anti-viral state in selective cell types.

Though IL-10 may influence the antiviral immune response via multiple pathways including via CCR2^+^ monocytes, our findings demonstrate that the effects of IL-10 are likely to be highly dependent on the location of IL-10 secreting T cells. Interestingly, nearly all IL-10-producing cells in infected reporter mice were amassed at the borders of viral lesions, rather than being more distributed throughout the inflamed, edematous tissue as were many virus-specific T cells (Fig 5). Likewise, most VV-specific IL-10gfp^+^ cells moved in restricted paths around keratinocytic foci of virus replication (Fig 6). This motility may play an important role in evenly distributing IL-10 around active viral lesions.

Due to the limitations of the IL10gfp^+^ reporter system used, we cannot, however, determine the extent to which IL-10 is secreted by circling *vs*. sessile IL-10gfp^+^ CD8^+^ T cells. Indeed, prior imaging studies of CD4^+^ T cells show that effector cytokine production is anti-correlative with movement [58, 59]. This uncertainty should be resolved with improved transgenic reporter systems that afford more precise measurement of ongoing IL-10 synthesis. Additionally, further study will be needed to carefully delineate the contributions of IL-10 generated by each T cell subset, as well as by those cells that are only minimally fluorescent in IL-10 reporter mice.

*In toto*, our findings uncover a unique aspect of local antiviral immunity with clear practical implications. With a critical role for IL-10-producing CD8^+^ T cells becoming evident for a number of widely different viruses, vaccines aimed at eliciting high numbers of multifunctional CD8^+^ T cells should consider adding IL-10 to the desired T cell-cytokine repertoire. Likewise, antiviral therapeutics that temporarily boost IL-10 levels in the tissue during acute infections may reduce viral burdens and hasten recovery, particularly if IL-10 can be delivered to the precise anatomical location where it is most effective.

## Materials and Methods

### Mice

Specific pathogen-free C57BL/6, B6(Cg)-Tyrc-2J/J, and IL-10gfp reporter transgenic mice on a C57BL/6 background were acquired from The Jackson Laboratory (B6.129S6-*Il10* ^*tm1Flv*^/J stock # 8379, “TIGER”, and B6(Cg)-Il10^tm1.1Karp^/J stock #14530, “Vert-X) and bred in house with B6(Cg)-Tyrc-2J/J (albino B6) for heterozygosity and homozygosity. 6–12-wk-old adult mice were used in all experiments. B6(Cg)-Tyrc-2J/J (albino B6) were also used where indicated. All mice were housed under specific pathogen–free conditions (including MNV, MPV, and MHV) and maintained on standard rodent chow and water supplied *ad libitum*.

For adoptive T cell transfer, spleens and nodes were removed from OT-I dsRed mice (as described in [26, 60]). Cells were purified to > 90% purity using an Automacs (Miltenyi Biotech) and using negative selection for CD8^+^ T cells. At least one day prior to infection, 1 x 10^5^ cells were transferred into IL-10gfp animals.

### Ethics statement

All animal experiments were conducted in accordance with the Animal Welfare Act and the recommendations in the Guide for the Care and Use of Laboratory Animals of the National Institutes of Health. NIAID animal facilities have full accreditation from the Association for Assessment and Accreditation of Laboratory Animal Care and are PHS-assured (Assurance Number: # A4149-01). All animal procedures were approved by the NIAID Animal Care and Use Committee.

### Viruses and infections

Mice were infected ec. in the ear pinnae by using a drop of 1x10^8^ PFU/mL rVV stock placed on the ear skin and gently poked a total of 10 times per ear with a bifurcated needle *(*similar to the human vaccination protocol). VVs used in this study were VV-Ova (full length ovalbumin protein containing SIINFEKL), VV-NP-S-GFP (with the influenza nucleoprotein (NP), SIINFEKL peptide (S), and green fluorescent protein; fluorescence is nuclear), VV-NP-S-BFP, and VV-BFP-Ub-SIINFEKL (cellular ubiquitin hydrolase liberates SIINFEKL peptide from the fusion protein). All have been previously described [26] and virus stocks were grown and titered in house.

### Flow cytometric analyses

Single cell suspensions of ears, cervical lymph nodes and spleens were prepared by collagenase digestion to liberate immune cells (Type I, Worthington Biochemicals) for 1 hr at 37°C and processed into single cell suspensions prior to filtration through 70 μm nylon cell strainers. The following antibodies were used to stain the cells: CD45 (clone 30-F11), CD8a (clone 53–6.7), CD8b (clone eBioH35-17.2), CD4 (clone RM4-5), CD11c (clone HL3), CD11b (clone M1/70), CD69 (clone H1.2F3), IL-10 (clone JES5-16E3), IFN-γ (clone XMG1.2), Granzyme B (clone NGZB), T-bet (clone eBio4B10). Antibodies were purchased from eBiosciences and BD Biosciences. CD8^+^ T cells were defined as CD8α^+^, CD8β^+^ cells (to exclude CD8α^+^ dendritic cells).

For intracellular cytokine staining, brefeldin A (10 μg/ml, Sigma-Aldrich) was added during collagenase digestion, and liberated cells were incubated an additional 3 hours in BFA-containing RPMI (Life Technologies) at 37°C. Cells were stained with 5 μg/mL of ethidium monoazide (EMA) for 15 min in the dark, exposed to light for 10 min to mark dead and dying cells. Cells were subsequently stained for the cell surface markers CD4, CD8, CD69, and CD45, fixed at room temperature for 30 min with eBiosciences FoxP3 Fix/Perm buffer, washed, then stained with anti-IFN-γ, anti-TNF-α, anti-Granzyme B, anti-T-bet and/or anti-FoxP3 in Perm buffer overnight at 4°C. Cells were analyzed on an LSR II flow cytometer (BD Biosciences) and resultant data analyzed using FlowJo (Treestar).

### Antibody administration

Unless otherwise indicated, mice received 0.25 mg of anti-IL-10 (clone JES5-2A5, BioXcell) intraperitoneally (i.p.) on day 5 and day 6 post infection.

### Viral titering via plaque assay

Ears were removed at various times p.i., collagenase digested for 1 hour at 37°C, and disrupted by vigorous pipetting. This suspension was freeze-thawed 3X, sonicated 3X, serially diluted and plated on TK^-/-^ cells. Cells were incubated for 2 days before counting the resulting plaques.

### qPCR cytokine array

Total RNA was isolated from whole ears using Trizol reagent (Invitrogen) according to the manufacturer’s instructions. cDNA was generated using RT^2^ first strand kit (Qiagen). Common cytokine PCR array (Qiagen) was used to quantitate cytokine mRNA fold increase over uninfected controls, according to the manufacturer’s instructions.

### VV genome quantification by qPCR

DNA was isolated from whole ears using the ArchivePure DNA Tissue Kit, (5 Prime, Gaithersburg MD) according to the manufacturer’s guidelines. Viral genomes were quantified by real-time PCR using primers specific for VV E3L gene. Primer sequences: 5′-GCAGAGATTGTGTGTGCGGCTATT-3′ and 5′-GGTGACAGGGTTAGCATCTTTCCA-3′ as previously reported [61]. Amplification of E3L and GAPDH were done in parallel using RT^2^ SYBR green qPCR Master Mix (SABiosciences). To quantify VV genome copies, a standard curve was generated using DNA from purified VV stock with a known PFU (determined by plaque assay). Viral copies were determined using a standard curve and were normalized to the amount of total input DNA.

### Intravital MPM imaging

MPM imaging was performed essentially as described previously [26, 62, 63]. Briefly, images were acquired on an upright Leica SP5 confocal microscope (Leica Microsystems) equipped with two Mai Tai Ti:Sapphire lasers (Spectra Physics) with 10-Watt pumps. Mice were anesthetized with avertin or isoflurane. Ears were immobilized on an imaging platform and bathed in warm saline; a 20x dipping objective (NA 1.00) was dipped in saline overlying ears for image acquisition. For imaging BFP/collagen/eGFP, imaging was performed in sequential mode with the first laser tuned to 900 nm and the second tuned to 800 nm for BFP excitation. Emitted fluorescence was collected with a four-channel non-descanned detector. For blue/green channels, wavelength separation was accomplished with a dichroic mirror at 495 nm followed by emission filters of 460/50 nm bandpass and 525/50 nm bandpass. For most movies (e.g. when time was critical), images were acquired using 2x zoom, using a 3-μm *z*-step for a total depth of 150 μm for 1 min intervals between series (needed to switch lasers). For 3D reconstructions, images were acquired at 1x zoom using 1024x1024 resolution and 1 μm z-steps for a total depth of 80–300 μms.

### Confocal imaging of frozen sections

Ears were fixed in PLP fixative (periodate-lysine-paraformaldehyde) overnight as reported in [64], cryoprotected in 15% sucrose, embedded in OCT medium (Electron Microscopy Sciences) and frozen in dry-ice cooled isopentane. Sixteen-micron sections were cut on a Leica cryostat (Leica Microsystems), blocked with 5% goat or donkey serum then stained with CD8 (53–6.7), anti-GFP (clone 5F12.4). Sections were incubated with secondary antibodies only as controls, and images were acquired using identical PMT (photomultiplier tube) and laser settings.

### Confocal tile scans

Scans were taken of entire infected ears equaling a 7 mm^2^ imaged area and individual fields (tiles) were merged into one image. Total image fluorescence was calculated using ImageJ software.

### MPM and confocal image analyses

For MPM analysis, maximum intensity projections (MIPs) were processed from z-stacks using Imaris (Bitplane). Because both collagen (second harmonic generation) and BFP appear in the blue channel, BFP expression was pseudocolored magenta. For tracking cellular movement, images were processed using a Gaussian filter, tracks were calculated using the “spot” function of Imaris. Average speeds were calculated using the spot detection function and the following parameters: autoregressive motion, gapclose 1, 7.5 μm object diameter, 20 μm maximum distance. Following automated analyses, tracks were analyzed individually for erroneous connections. Track straightness was calculated according to the standard convention of displacement/track length.

### Statistical analyses

Significances were calculated using GraphPad (Prism) using unpaired two-tailed Student’s *t* test.

## Supporting Information

S1 Movie IL-10gfp^+^ T cells localize around VV-infected foci. Three-dimensional rotation of a maximum intensity projection (MIP) of an MPM image of an IL-10gfp reporter mice (TIGER) 6 d.p.i. with VV NP-S-Blue fluorescent protein (BFP, pseudocolored magenta). IL-10gfp^+^ cells and autofluorescent hairs = green. Collagen (dermis) = blue. Scale bars = μm. See also Fig 4.
(MOV)

S2 Movie IL-10gfp^+^ T cells are closely associated with VV-infected foci. Three-dimensional rotation of a maximum intensity projection (MIP) of an MPM image of an IL-10gfp-reporter mice (TIGER) that received 1 x 10^5^ adoptively transferred dsRed^+^ OT-I CD8^+^ T cells (red) before infection. (OT-I T cells are all antigen specific for SIINFEKL encoded by VV-BFP-ub-SIINFEKL.) Image was acquired 6 d.p.i. Virus-infected cells = pseudocolored magenta. IL-10gfp^+^ cells and autofluorescent hairs = green. Collagen (dermis) = blue. Scale bars = μm. See also Fig 5.
(MOV)

S3 Movie Relatively few IL-10gfp^+^ T cells are located in areas lacking virus-infected cells. Three-dimensional rotation of a maximum intensity projection (MIP) of an MPM image of an IL-10gfp-reporter mice (TIGER) that received 1 x 10^5^ adoptively transferred dsRed^+^ OT-I CD8^+^ T cells (red) before infection. (OT-I T cells are all antigen specific for SIINFEKL encoded by VV-BFP-ub-SIINFEKL.) Image was acquired in an area that lacked virus-driven fluorescent protein expression (which would be magenta) on 6 d.p.i. IL-10gfp^+^ cells and autofluorescent hairs = green. Collagen (dermis) = blue. Scale bars = μm. See also Fig 5.
(MOV)

S4 Movie IL-10gfp^+^ T cells mobile in and around viral foci of infection. Movie of MIP images taken 6 d.p.i. with VV NP-S-BFP (pseudocolored magenta) over a 2 hr imaging session in an IL-10gfp-reporter mice (TIGER) that received 1 x 10^5^ adoptively transferred dsRed^+^ OT-I CD8^+^ T cells (red) before infection. (OT-I T cells are all antigen specific for SIINFEKL encoded by VV-BFP-ub-SIINFEKL.) Image was acquired on 6 d.p.i. Virus-infected cells = pseudocolored magenta. IL-10gfp^+^ cells and autofluorescent hairs = green. Collagen (dermis) = blue. Scale bars = μm. Time = min. See also Fig 6.
(MOV)

S5 Movie IL-10gfp^+^ T cells are mobile in and around viral foci of infection. Movie of MIP images taken 6 d.p.i. with VV NP-S-BFP (pseudocolored magenta) over a 2 hr imaging session in an IL-10gfp-reporter mice (TIGER) that received 1 x 10^5^ adoptively transferred dsRed^+^ OT-I CD8^+^ T cells (red) before infection. (OT-I T cells are all antigen specific for SIINFEKL encoded by VV-BFP-ub-SIINFEKL.) Image was acquired on 6 d.p.i. Virus-infected cells = pseudocolored magenta. IL-10gfp^+^ cells and autofluorescent hairs = green. Collagen (dermis) = blue. Scale bars = μm. Time = min. See also Fig 6.
(MOV)

S6 Movie IL-10gfp^+^ CD8^+^ T cells are mobile around VV lesions. Movie of MIP images taken 6 d.p.i. with VV-NP-S-BFP (pseudocolored magenta) over a 20 min. imaging session without adoptive transfer of other T cells. IL-10gfp^+^ cells and autofluorescent hairs = green. Collagen (dermis) = blue. Time = min. Scale bars = μm. See also Fig 6.
(MOV)

S7 Movie Mobile CCR2rfp^+^ monocytes can be infected with VV. Movie of MIP images taken 6 d.p.i. with VV-NP-S-GFP (green) over an hr imaging session in an CCR2rfp^+^/^-^ mouse cells (red CCR2^+^ monocytes). Image was acquired 7 d.p.i. Movement of one virus-infected monocytes is highlighted with a circle. Collagen (dermis) = blue. Scale bars = μm. Time = min. See also Fig 7.
(MOV)

S1 Fig CD4^+^ and CD8^+^ T cells produce IL-10 protein after epicutaneous vaccinia virus infection. A) Percentage of CD4^+^ T cells isolated from the skin producing IL-10 (determined by antibody staining for intracellular protein) on days 6, 12, and 13 post-infection with recombinant vaccinia virus expressing ovalbumin (Vac-Ova). White bars = cells analyzed directly *ex vivo*. Black bars = cells that were restimulated for 5 hours with Vac-Ova. Pseudocolored dot plots for an individual animal on day 6 post-infection are shown on the right. IL-10 staining is on the x-axis. B) as in A) except gating on CD8^+^ T cells. N = 3 mice/group. Error bars = SEM.
(TIF)

S2 Fig IL-10gfp^+^ CD8^+^ T cells in VV-infected skin are not regulatory T cells on day 6 post-infection. Flow cytometric dot plot of single cell suspensions of ears 6 days post-VV-infection. Cells were gated on CD45^+^ cells, then on CD8^+^ T cells, then on CD8^+^ and FoxP3^+^ cells (stained intracellularly using a kit from eBioscience). Gate shows the percentage of FoxP3^+^ CD8^+^ T cells on day 6.
(TIF)

S3 Fig Mobility of dermal IL-10gfp^+^ cells on day 6 post-VV infection. A) Average cellular speeds of IL-10gfp^+^ cells over 20 min imaging periods. Dots represent individual cells; groups represent different experiments. Means of average speeds are shown with a black bar. B) IL-10gfp^+^ cell track straightness (track displacement/track length) C) Mean track length of IL-10gfp^+^ cells over 20 min. D) Percentage of IL-10gfp^+^ cells that were stopped or slowly motile, moving at average speeds less than 2.5 μm/min. Dots show the percentage of each of 8 experiments.
(TIF)

S4 Fig Characterization of CCR2^+^ monocytes in VV-infected ears of CCR2rfp ^+^/^+^, ^+^/^-^, and ^-^/^-^ mice. Monocyte populations in CCR2rfp null, heterozygous, and homozygous mice 7 days post-VV-infection. A) Flow cytometric plots of single cell suspensions generated from infected ears. Cells were gated on CD45^+^ leukocytes, then on CCR2^+^ CD11b^+^ cells. (note: CCR2 was examined by cell-surface antibody staining due to lack of rfp detection on our cytometer) B) Numbers of CCR2^+^ monocytes per ear on day 7 post-infection. We selected heterozygous mice for further analysis because of the reduction in number of monocytes (similar to the effect of IL-10 treatment).
(TIF)

S1 Data Supplemental data. (DOCX)
