# Supplementary figures and images for: Locally Produced IL-10 Limits Cutaneous Vaccinia Virus Spread
Source: PLoS Pathog. 2016 Mar 18;12(3):e1005493. doi: 10.1371/journal.ppat.1005493 (PMC4798720; doi:10.1371/journal.ppat.1005493)

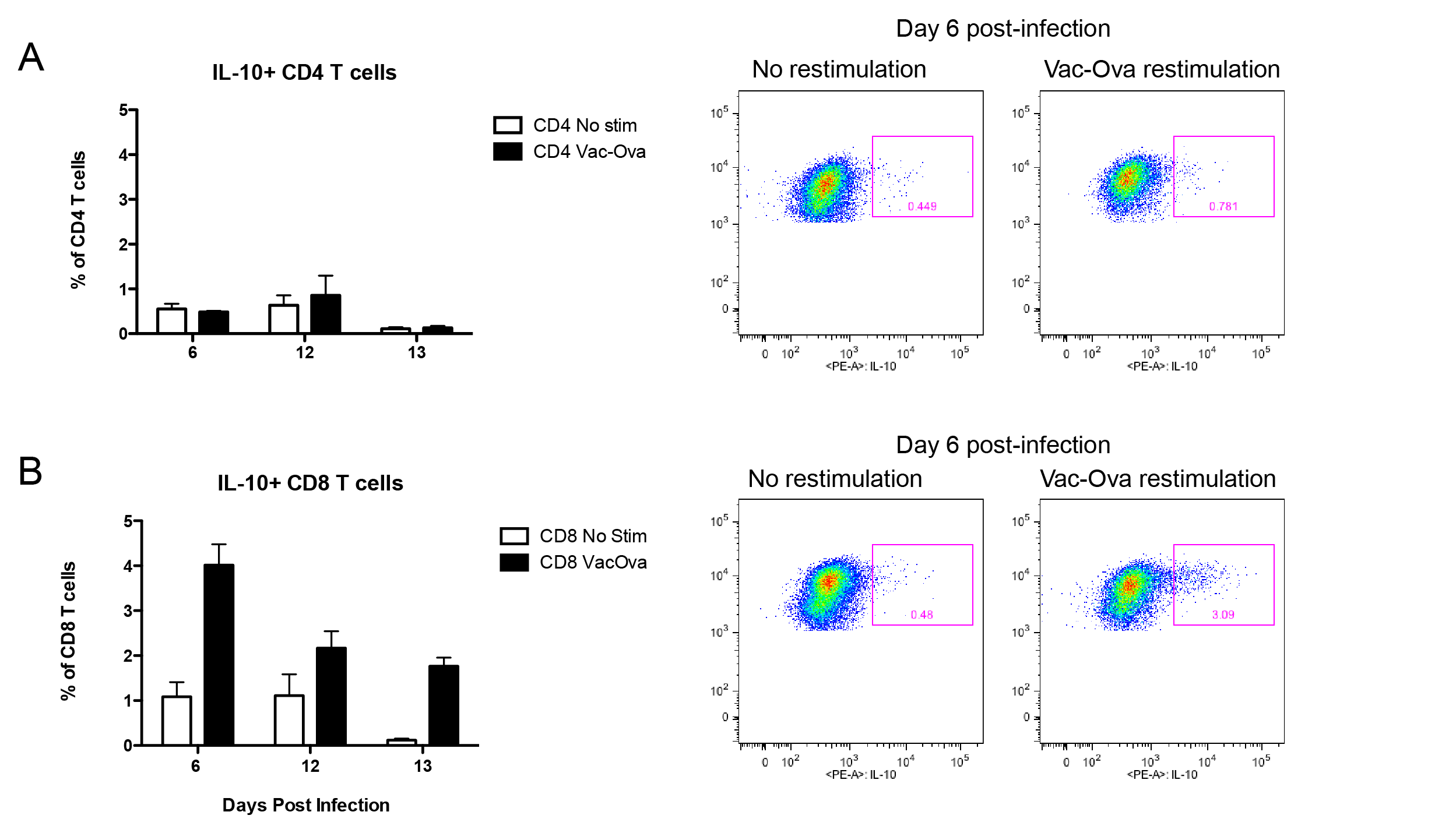

Supplement: S1 Fig — A) Percentage of CD4+ T cells isolated from the skin producing IL-10 (determined by antibody staining for intracellular protein) on days 6, 12, and 13 post-infection with recombinant vaccinia virus expressing ovalbumin (Vac-Ova). White bars = cells analyzed directly ex vivo. Black bars = cells that were restimulated for 5 hours with Vac-Ova. Pseudocolored dot plots for an individual animal on day 6 post-infection are shown on the right. IL-10 staining is on the x-axis. B) as in A) except gating on CD8+ T cells. N = 3 mice/group. Error bars = SEM. (TIF) [file ppat.1005493.s008.tif]

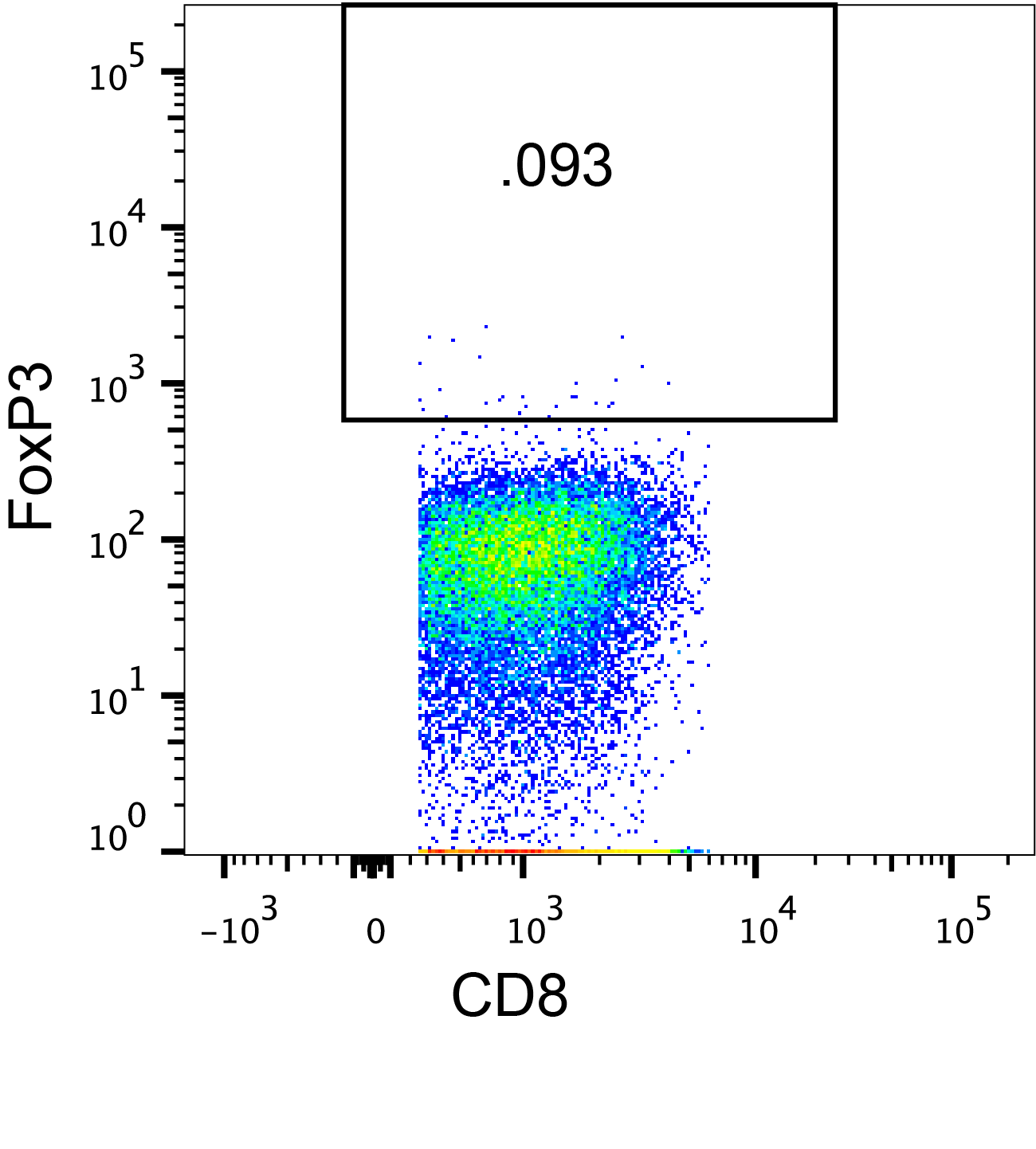

Supplement: S2 Fig — Flow cytometric dot plot of single cell suspensions of ears 6 days post-VV-infection. Cells were gated on CD45+ cells, then on CD8+ T cells, then on CD8+ and FoxP3+ cells (stained intracellularly using a kit from eBioscience). Gate shows the percentage of FoxP3+ CD8+ T cells on day 6. (TIF) [file ppat.1005493.s009.tif]

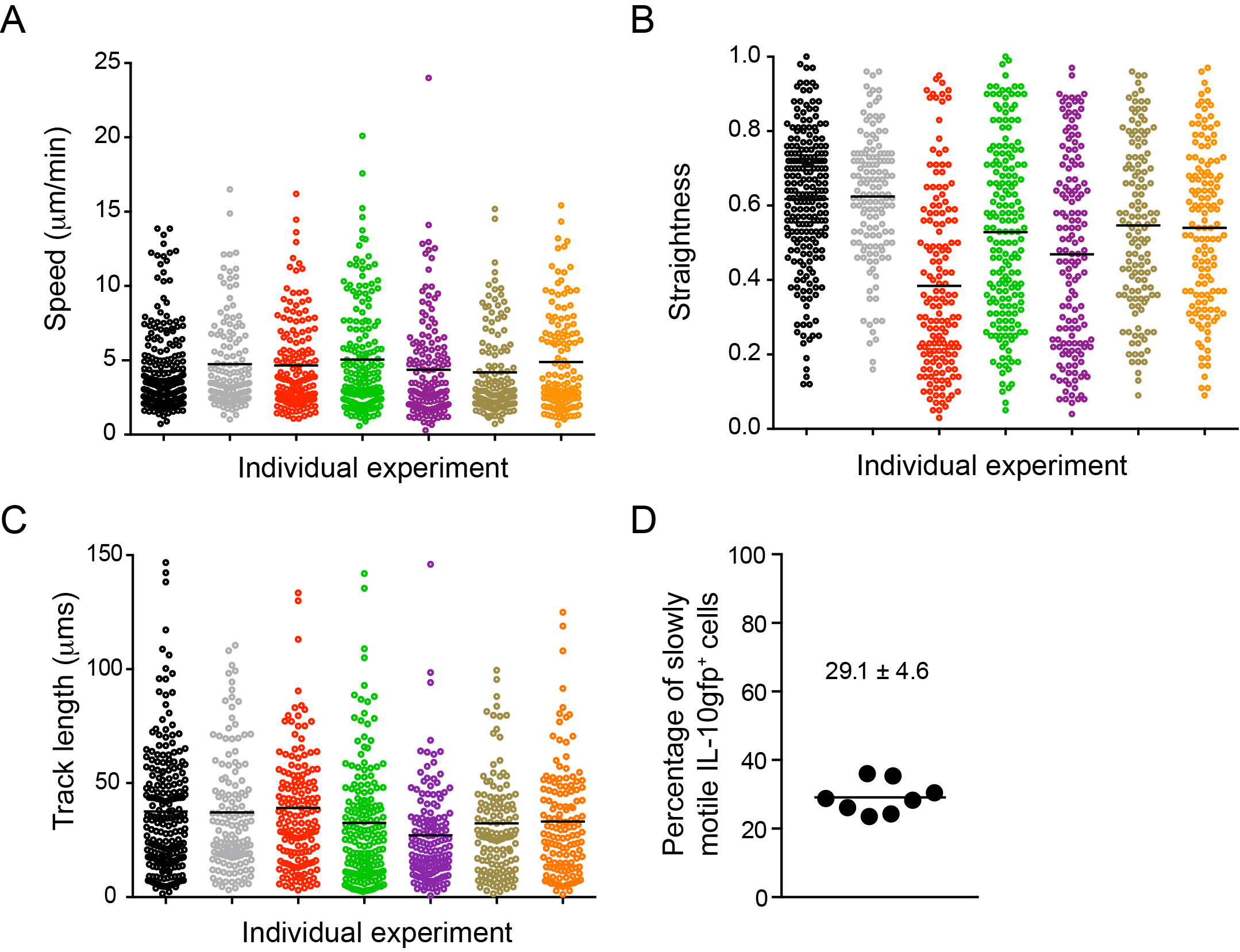

Supplement: S3 Fig — A) Average cellular speeds of IL-10gfp+ cells over 20 min imaging periods. Dots represent individual cells; groups represent different experiments. Means of average speeds are shown with a black bar. B) IL-10gfp+ cell track straightness (track displacement/track length) C) Mean track length of IL-10gfp+ cells over 20 min. D) Percentage of IL-10gfp+ cells that were stopped or slowly motile, moving at average speeds less than 2.5 μm/min. Dots show the percentage of each of 8 experiments. (TIF) [file ppat.1005493.s010.tif]

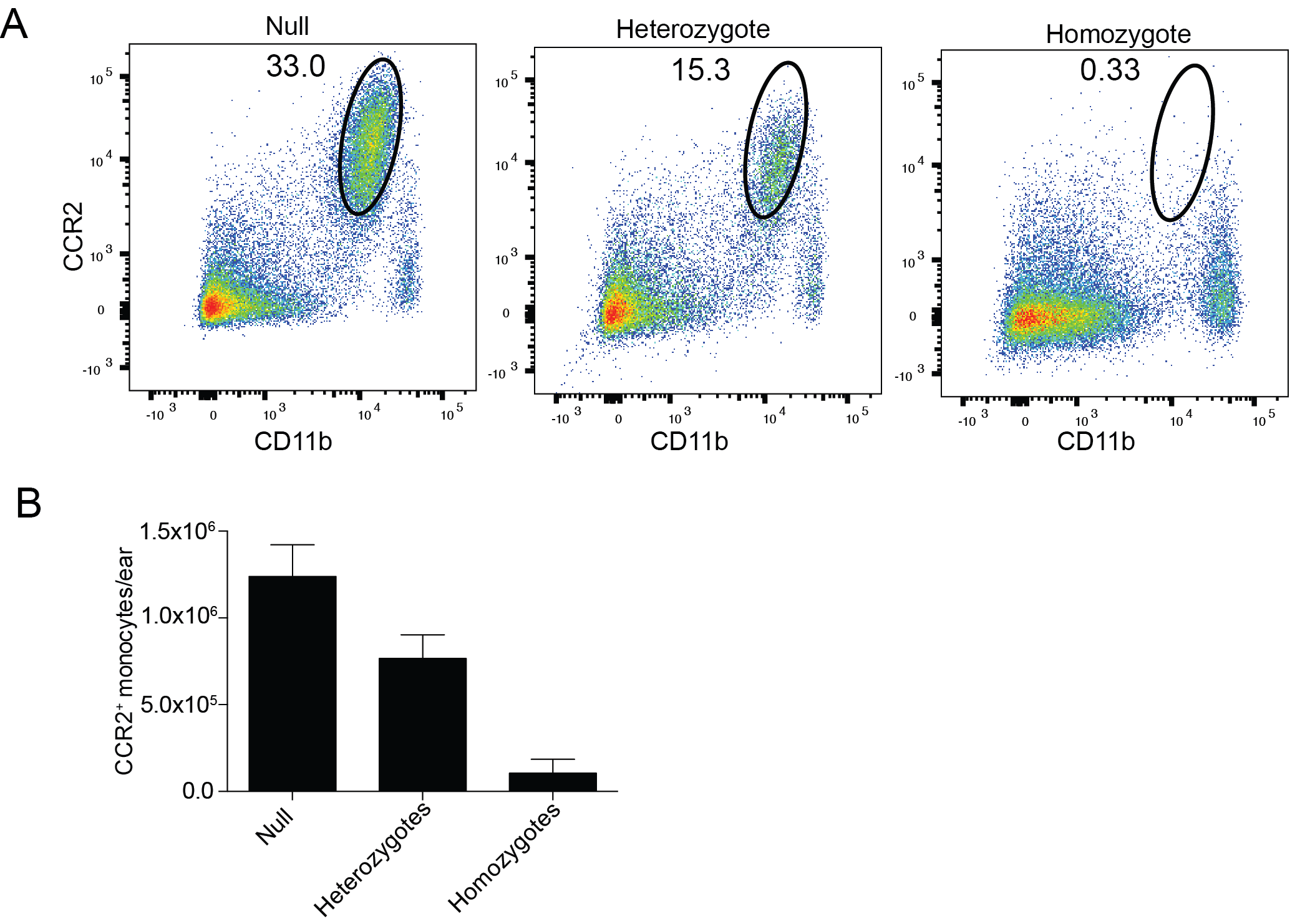

Supplement: S4 Fig — Monocyte populations in CCR2rfp null, heterozygous, and homozygous mice 7 days post-VV-infection. A) Flow cytometric plots of single cell suspensions generated from infected ears. Cells were gated on CD45+ leukocytes, then on CCR2+ CD11b+ cells. (note: CCR2 was examined by cell-surface antibody staining due to lack of rfp detection on our cytometer) B) Numbers of CCR2+ monocytes per ear on day 7 post-infection. We selected heterozygous mice for further analysis because of the reduction in number of monocytes (similar to the effect of IL-10 treatment). (TIF) [file ppat.1005493.s011.tif]
