# Supplementary material for: Locally Produced IL-10 Limits Cutaneous Vaccinia Virus Spread
Source: PLoS Pathog. 2016 Mar 18;12(3):e1005493. doi: 10.1371/journal.ppat.1005493 (PMC4798720; doi:10.1371/journal.ppat.1005493)
Supplement: S1 Data — (DOCX) [file ppat.1005493.s012.docx]

Supplemental Data


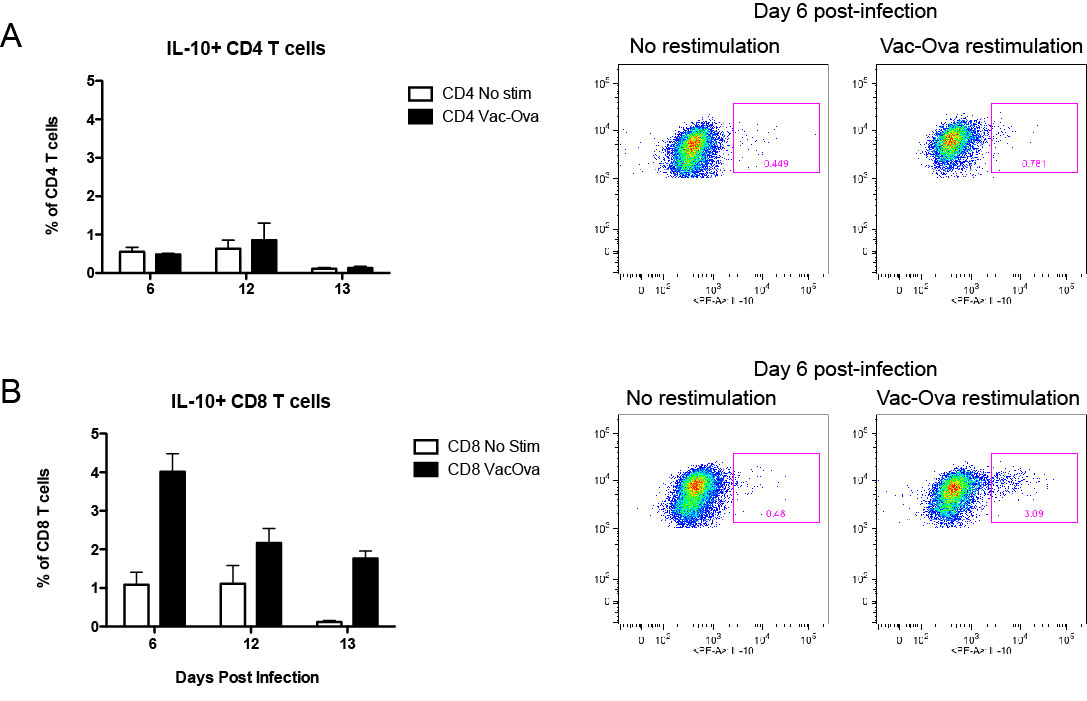


**Supplemental Figure 1. CD4^+^ and CD8^+^ T cells produce IL-10 protein after epicutaneous vaccinia infection.**

A) Percentage of CD4^+^ T cells isolated from the skin producing IL-10 (determined by antibody staining for intracellular protein) on days 6, 12, and 13 post-infection with recombinant vaccinia virus expressing ovalbumin (Vac-Ova). White bars = cells analyzed directly *ex vivo*. Black bars = cells that were restimulated for 5 hours with Vac-Ova. Pseudocolored dot plots for an individual animal on day 6 post-infection are shown on the right. IL-10 staining is on the x-axis. B) as in A) except gating on CD8^+^ T cells. N= 3 mice/group. Error bars = SEM.


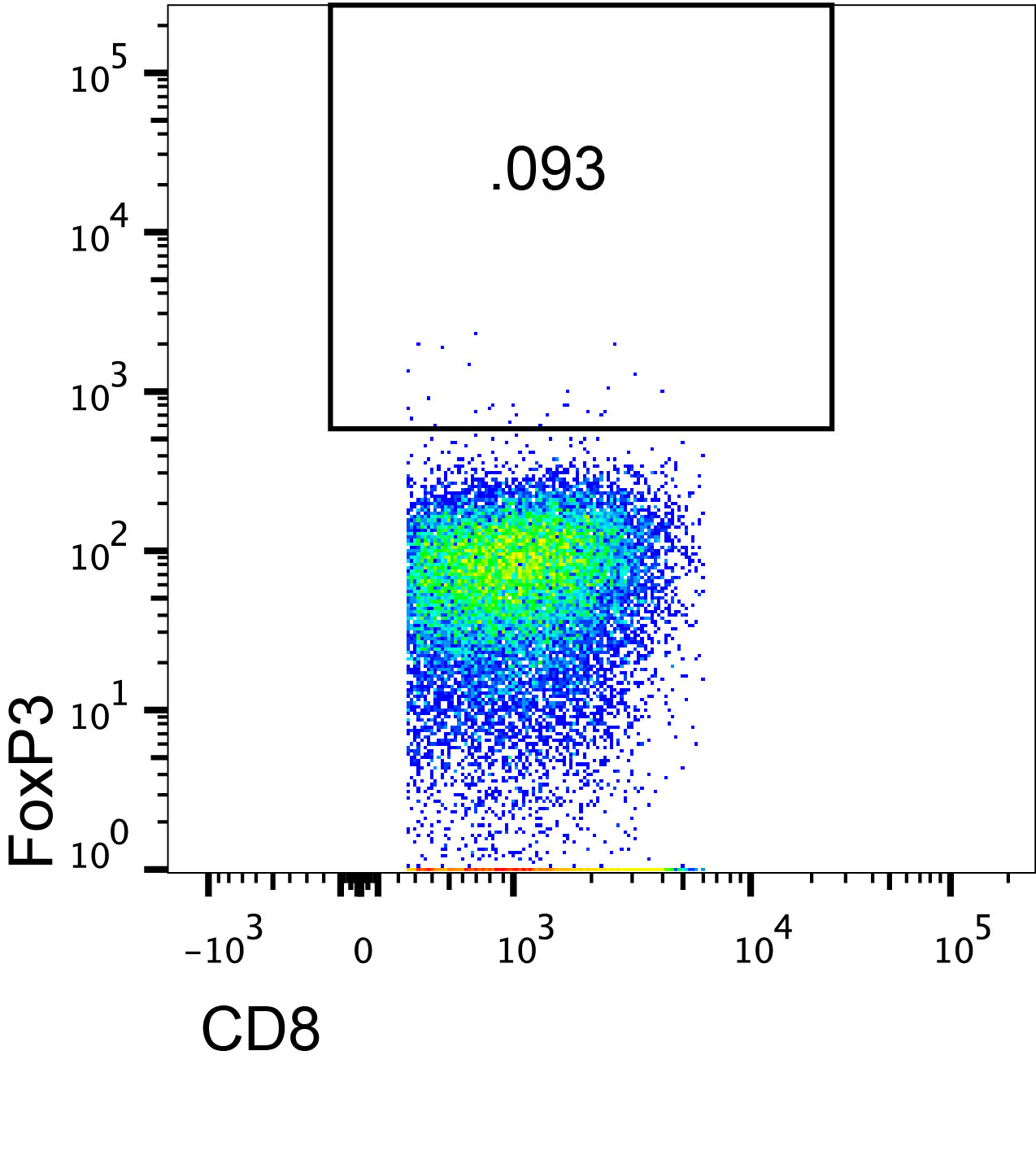
**Supplemental Figure 2. IL-10gfp^+^ CD8^+^ T cells in VV-infected skin are not regulatory T cells on day 6 post-infection.**

Flow cytometric dot plot of single cell suspensions of ears 6 days post-VV-infection. Cells were gated on CD45^+^ cells, then on CD8^+^ T cells, then on CD8^+^ and FoxP3^+^ cells (stained intracellularly using a kit from eBioscience). Gate shows the percentage of FoxP3^+^ CD8^+^ T cells on day 6.


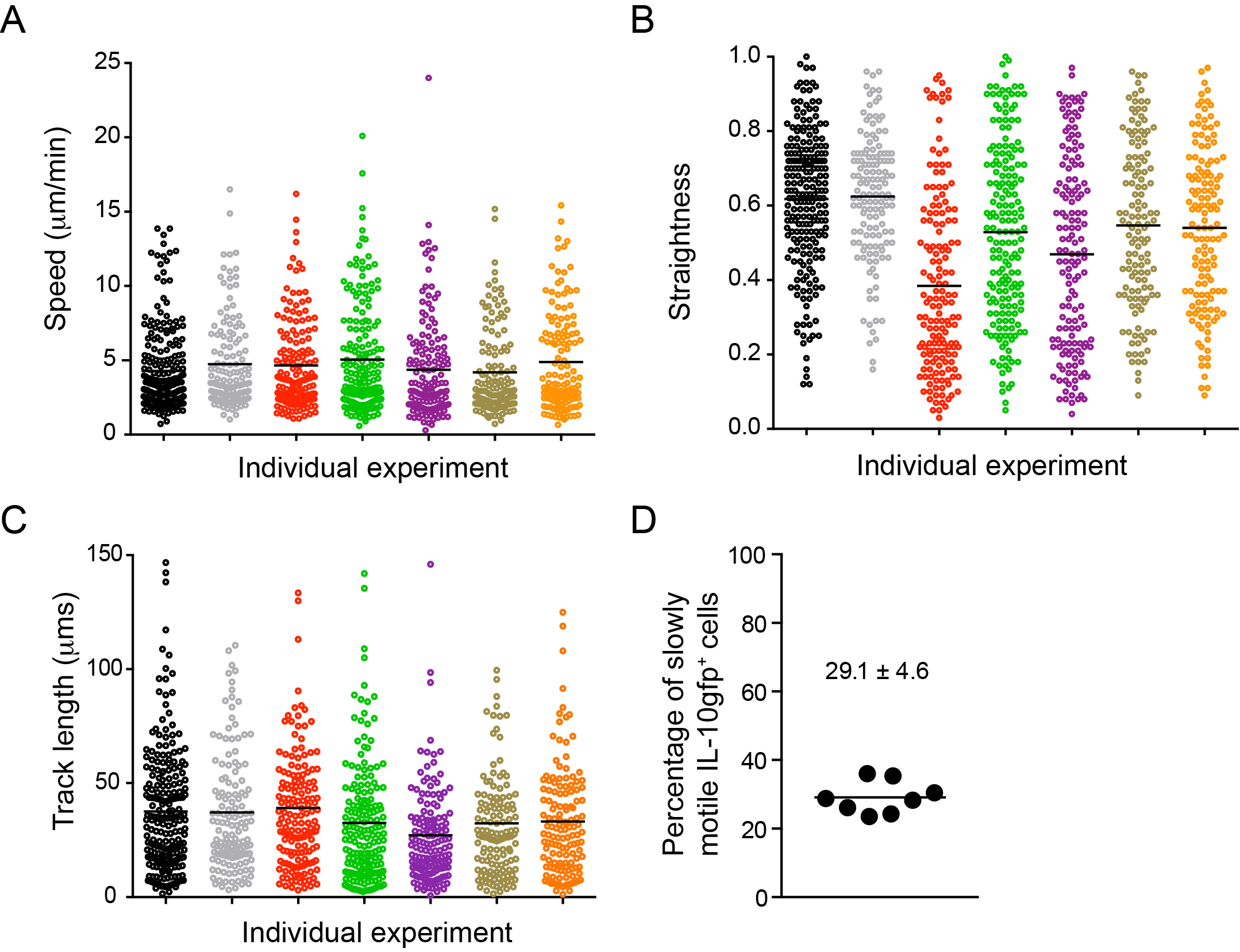


Supplemental Figure 3. Mobility of dermal IL-10gfp^+^ cells on day 6 post-VV infection.

Average cellular speeds of IL-10gfp^+^ cells over 20 min imaging periods. Dots represent individual cells; groups represent different experiments. Means of average speeds are shown with a black bar. **B)** IL-10gfp^+^ cell track straightness (track displacement/track length) **C)** Mean track length of IL-10gfp^+^ cells over 20 min. **D)** Percentage of IL-10gfp+ cells that were stopped or slowly motile, moving at average speeds less than 2.5 μM/min. Dots show the percentage of each of 8 experiments.


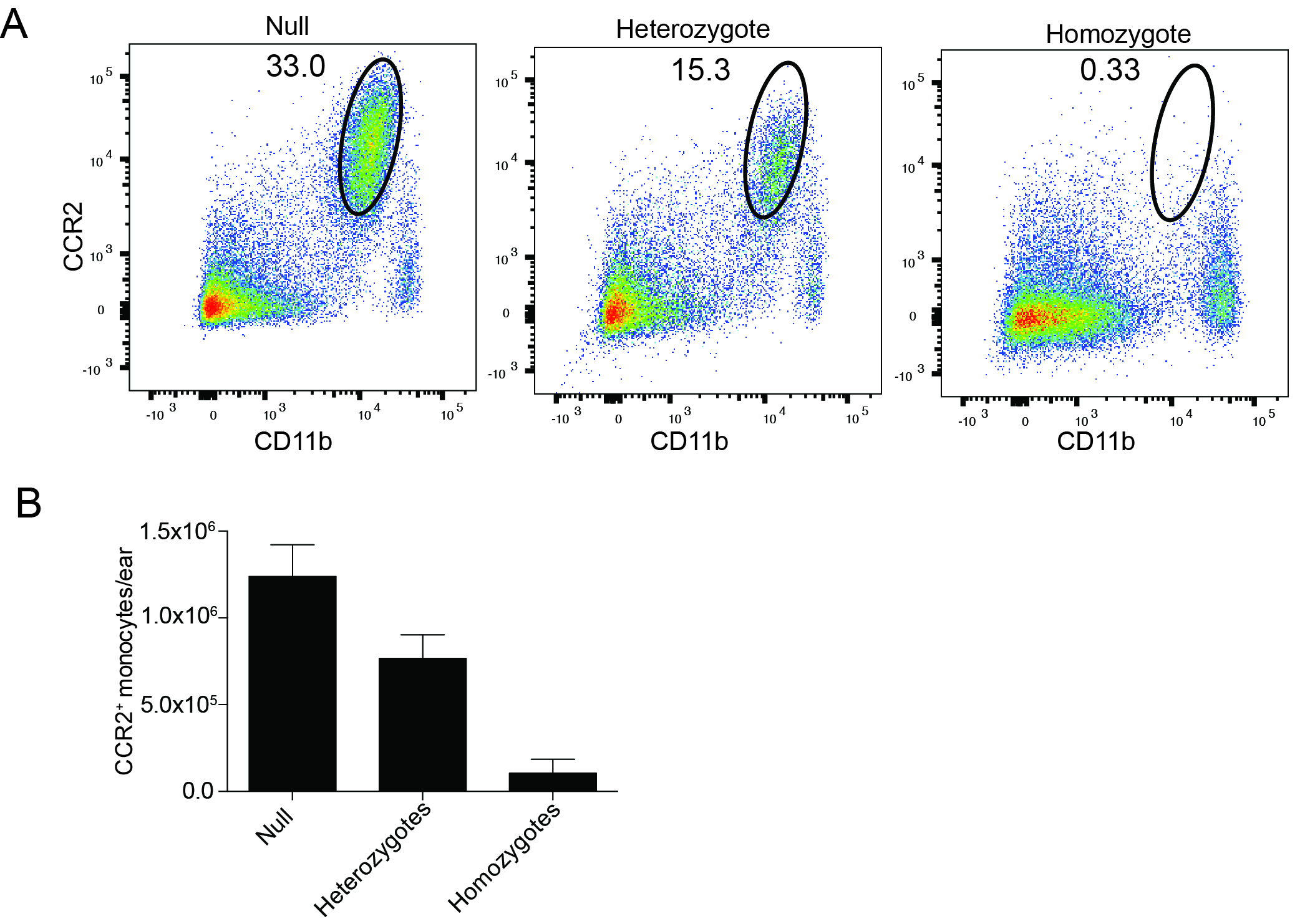


Supplemental Figure 4. Characterization of CCR2^+^ monocytes in VV-infected ears of CCR2rfp ^+^/^+^, ^+^/^-^, and ^-^/^-^ mice.

Monocyte populations in CCR2rfp null, heterozygous, and homozygous mice 7 days post-VV-infection. **A**) Flow cytometric plots of single cell suspensions generated from infected ears. Cells were gated on CD45^+^ leukocytes, then on CCR2^+^ CD11b^+^ cells. (note: CCR2 was examined by cell-surface antibody staining due to lack of rfp detection on our cytometer) **B)** Numbers of CCR2^+^ monocytes per ear on day 7 post-infection. We selected heterozygous mice for further analysis because of the reduction in number of monocytes (similar to the effect of IL-10 treatment).
